# Enlight: A Comprehensive Quality and Therapeutic Potential Evaluation Tool for Mobile and Web-Based eHealth Interventions

**DOI:** 10.2196/jmir.7270

**Published:** 2017-03-21

**Authors:** Amit Baumel, Keren Faber, Nandita Mathur, John M Kane, Fred Muench

**Affiliations:** ^1^ Psychiatry Research The Feinstein Institute for Medical Research Glen Oaks, NY United States; ^2^ Northwell Hofstra School of Medicine Hempstead, NY United States

**Keywords:** eHealth, mHealth, assessment, evaluation, quality, persuasive design, behavior change, therapeutic alliance

## Abstract

**Background:**

Studies of criteria-based assessment tools have demonstrated the feasibility of objectively evaluating eHealth interventions independent of empirical testing. However, current tools have not included some quality constructs associated with intervention outcome, such as persuasive design, behavior change, or therapeutic alliance. In addition, the generalizability of such tools has not been explicitly examined.

**Objective:**

The aim is to introduce the development and further analysis of the Enlight suite of measures, developed to incorporate the aforementioned concepts and address generalizability aspects.

**Methods:**

As a first step, a comprehensive systematic review was performed to identify relevant quality rating criteria in line with the PRISMA statement. These criteria were then categorized to create Enlight. The second step involved testing Enlight on 42 mobile apps and 42 Web-based programs (delivery mediums) targeting modifiable behaviors related to medical illness or mental health (clinical aims).

**Results:**

A total of 476 criteria from 99 identified sources were used to build Enlight. The rating measures were divided into two sections: quality assessments and checklists. Quality assessments included usability, visual design, user engagement, content, therapeutic persuasiveness, therapeutic alliance, and general subjective evaluation. The checklists included credibility, privacy explanation, basic security, and evidence-based program ranking. The quality constructs exhibited excellent interrater reliability (intraclass correlations=.77-.98, median .91) and internal consistency (Cronbach alphas=.83-.90, median .88), with similar results when separated into delivery mediums or clinical aims. Conditional probability analysis revealed that 100% of the programs that received a score of fair or above (≥3.0) in therapeutic persuasiveness or therapeutic alliance received the same range of scores in user engagement and content—a pattern that did not appear in the opposite direction. Preliminary concurrent validity analysis pointed to positive correlations of combined quality scores with selected variables. The combined score that did not include therapeutic persuasiveness and therapeutic alliance descriptively underperformed the other combined scores.

**Conclusions:**

This paper provides empirical evidence supporting the importance of persuasive design and therapeutic alliance within the context of a program’s evaluation. Reliability metrics and preliminary concurrent validity analysis indicate the potential of Enlight in examining eHealth programs regardless of delivery mediums and clinical aims.

## Introduction

The wide distribution of personal digital devices has changed the potential to dramatically enhance public access to health interventions; tens of thousands of health, wellness, and medical apps are now available for download from online stores [1]. From a public health perspective, the large number of available eHealth intervention programs makes it impossible to empirically evaluate them using traditional research methods. In addition, the time-consuming and costly process of product evaluation may result in the technology investigated in the trial becoming obsolete by the time the results are published [2] and may also make it difficult for health system leaders to engage with potential vendors [3,4]. Agile science tries to answer some of these challenges by focusing on an adaptable and nimble scientific process to support the collective development and evaluation of interventions [5], while taking into account the rapid pace of change in the technologies that support digital interventions [6]. However, despite developments in research methods, patients and medical professionals can browse through the Web or access mobile app stores to download and use the large number of available and unexamined programs. To complicate things further, the settings in which such programs are being utilized are different than within studies where participants are being proactively recruited, paid for filling out assessments, and have scheduled check-in appointments. As a result, some of the support that participants receive in studies does not translate to the real world, which might impact intervention outcomes. Unfortunately, existing systems of user-based ratings that collect assessments in the real world are not designed to offer a metric of medical appropriateness, safety, or effectiveness [7].

Criteria-based rating scales have been developed to address this evaluation challenge. These scales are then used by trained raters to objectively examine and score the quality of eHealth intervention programs based on core concepts, each comprised of different criteria [8-11]. The importance of using a clearly defined rating system is strengthened by the fact that without the use of such systems scoring tends not to be highly reliable [7]. In facilitating the cost-effective evaluation of available eHealth interventions, such tools can enable stakeholders to discuss programs’ potential prior to empirical testing [4] and to provide information that supports user recommender systems [12-14]. Although these tools have several potential uses, we will relate to two aspects we believe to be important for the contributions of these tools to the evaluation of eHealth interventions that were not covered previously: examining quality domains that relate to programs’ therapeutic potential and tool generalizability.

### Quality Rating Domains Related to Programs’ Therapeutic Potential

#### Persuasive Design and Behavior Change Principles

Persuasive design aims at understanding what influences people’s behavior and decision making, and then uses this information to design compelling user interactions [15,16]. Interestingly, no previous rating scale aiming to evaluate the quality of eHealth intervention programs using different concepts has related to persuasive design or behavior change quality criteria, even though such concepts have been shown to be important in the evaluation of eHealth interventions potential. For example, Kientz et al [17] compared the performance of Nielsen and Molich’s usability heuristics [18] to persuasion and demonstrated that persuasion heuristics enabled the identification of more severe and more relevant interface problems in terms of persuasive, cultural, and informational issues. Kelders et al [19] showed that elements of persuasive design uniquely explained the variance in adherence to eHealth Web-based interventions, and Webb et al [20] showed that eHealth interventions that better incorporated behavior change theories also tended to have larger effects in increasing positive health-related behaviors. Altogether, these studies suggest that the quality of program persuasive design directly impacts its therapeutic potential and therefore has to be addressed when evaluating these programs.

#### Therapeutic Alliance Principles

No previous quality rating scale has directly assessed the therapeutic alliance being nurtured by the eHealth intervention program. Overall, studies focusing on nontechnological interventions have shown that the therapeutic alliance is one of the most robust measures for predicting psychotherapy success (eg, [21-23]) and suggested its promise for predicting intervention quality in the medical domain (eg, [24-27]). However, evaluating the potential therapeutic alliance between users and software programs requires the reexamination and adaptation of the original concept, which applies to a therapeutic relationship between people.

Studies suggest that therapeutic alliances with eHealth intervention programs do exist and that such alliances may play a role in increasing the adherence to [28,29] and effectiveness [30] of these programs. Scholars have indicated the variance in relational factors embedded within eHealth interventions that makes some programs better at nurturing a therapeutic alliance with their users [31-33]. Although these examinations are currently of a preliminary nature, there is a need for a standardized assessment measure that can adequately capture the concept of an e-therapeutic alliance and its place in the overall picture of product quality.

Overall, there is a need to develop standardized operational definitions to assess the quality of all aspects of eHealth intervention programs [34], including those relating to therapeutic potential [4]. To best address the unique contribution of each quality domain, the complete scope of different criteria should be taken into account. Such assessment will also enable stakeholders to investigate the interactions between different quality domains and their impact on outcomes.

### Tool Generalizability

Another aspect that has yet to be fully examined is whether criteria-based rating tools may enable us to reliably rate eHealth intervention programs that are developed and designed to be used in different delivery mediums (eg, mobile, personal computer, other). Such reliable rating would enable examination of programs leveraging more than one delivery medium and comparison between the qualities of programs regardless of their delivery mediums. Recently, Gomez Quiñonez et al [35] demonstrated that a Web-based app targeting adult physical activity provided better or similar results compared to the same mHealth app. Subsequently, in a recent systematic review of digital parent training programs, only one mobile app was identified, which was used as an adjunct to treatment, whereas most computer programs were used as a standalone intervention [36]. It might be that, for certain treatment aims, different design aspects limit the potential of one delivery medium, which could be examined when using the same framework of evaluation. The nature of quality criteria is that they relate to broad principles in terms of product design, which facilitates their use in different contexts [37]. The remaining question in terms of a tool’s generalizability is focused on the ability to use the same standardized measure to evaluate programs targeting different clinical aims (eg, behaviors related to medical conditions, mental health). During the development of previous eHealth-related rating scales, the reliability matrix of ratings based on different clinical aims was not demonstrated. It is important to pay attention to generalizability during the tool development phase because the development of scoring benchmarks might be influenced by the nature of reviewed programs.

### Study Aims

The gaps in the literature laid the foundation for the development of Enlight, a suite of criteria-based measurements aimed at enabling scholars to objectively rate eHealth interventions based on different quality concepts regardless of their delivery medium or clinical aims. Therefore, the aims of this study were to (1) identify relevant published criteria through a comprehensive systematic review that also addresses quality criteria related to persuasive design, behavior change, and therapeutic alliance/principles (a systematic review that relates to different aspects of eHealth and mHealth interventions including all concepts mentioned was not incorporated before and would enable the establishment of the tool based on a comprehensive examination of the current know-how in this field); (2) develop Enlight, a suite of criteria-based quality measures related to separate aspects of eHealth programs; (3) establish the measures’ reliability and generalizability in evaluating different delivery mediums and clinical aims; and (d) examine the intercorrelations between different quality constructs and between them and preliminary validity measures.

## Methods

This study was completed in two parts, each reflecting different procedures used. The first part, “Enlight Development,” included a systematic search for quality criteria, the classification of these criteria into core domains and subcategories, and the creation of the different scales. The second part involved reliability testing and further analysis of the interrelationships between the quality constructs and correlations with preliminary concurrent validity measures. As emphasized, we examined the results with respect to different delivery mediums and clinical aims.

### Enlight Development

#### Systematic Review and Collection of Quality Criteria

The systematic review was carried out in line with the Preferred Reporting Items for Systematic Reviews and Meta-Analyses (PRISMA) statement guidelines [38] (see Multimedia Appendix 1 for a complete list of PsycINFO database search terms used). We conducted comprehensive computer searches of IEEE Xplore, PsycINFO, PubMed, and Science Direct databases for English articles published between January 1, 2000 and April 8, 2016, containing explicit Web- or mobile app-based quality criteria. The search time window was limited to 2000 due to rapid developments in technology [2] and to reflect technologies that largely meet the expectations of today’s users [4]. For general quality criteria, we searched for papers in varying combinations for criteria (eg, “criteria” OR “principle*”), assessments (eg, “assess*” OR “measur*”), and delivery mediums (eg, “mobile*” OR “Web*”). We also searched for papers in varying combinations for criteria (eg, “criteria” OR “principle*”) specifically related to persuasive design and behavior change. A manual search for additional references was conducted by examining the reference lists of identified papers and previous review articles. We also reviewed grey literature by Google searching, looking into key websites (eg, Nielsen Norman Group), and asking experts for recommendations. To identify English articles containing explicit quality rating criteria in terms of therapeutic alliance/principles, we conducted comprehensive computer searches of PsycINFO, PubMed, and Science Direct databases for articles published by April 8, 2016. We searched for papers related to quality criteria (eg, “potential” OR “criteria” OR “principle*”) in the field of psychotherapy and for papers related to therapeutic alliance questionnaires.

#### Data Extraction and Categorization

Following the extraction of criteria from identified sources, we established a multidisciplinary advisory team to support the classification of these criteria into core domains and subcategories, and to support the development of the measures’ items and categories (see Multimedia Appendix 2, Advisory Team). Because the criteria identified for therapeutic alliance ratings were not focused on eHealth interventions (but rather on a human therapist), a thematic analysis [39] was conducted to redefine these criteria in terms of eHealth interventions as a preliminary step before categorizing them and building the final scale. This step was carried out by three licensed clinical psychologists.

### Enlight Testing

#### Identifying Relevant eHealth Intervention Programs

To establish the generalizability and transferability of the tool, we tested Enlight on programs targeting either modifiable behaviors related to chronic medical illnesses (ie, health-related behaviors) or mental health, and on programs delivered through mobile apps or websites (accessed through a personal computer). The systematic identification of relevant programs followed the PRISMA statement guidelines [38] (see Multimedia Appendix 3 for search terms used to identify free eHealth intervention programs). For health-related behaviors, we targeted behaviors considered to be among the leading preventable causes of death due to chronic medical conditions in the United States [40]: diet, physical activity, smoking cessation, and alcohol cessation. For mental health, we focused our search on the terms depression, anxiety, mental health, and well-being. For both websites and mobile apps, we included only free-to-use programs aimed at the specific condition that were published in English.

To identify relevant mobile apps, we conducted a systematic search of the Google Play store on September 5, 2016, by using search terms relevant for each condition (eg, diet: diet or weight loss). The inclusion criteria for mobile apps were (1) English language, (2) free of charge, and (3) from Android categories “Health & Fitness” and “Medical” following a careful examination of program type in different Android categories. To identify relevant Web-based programs, a systematic search was conducted on September 5, 2016, using a Google Search query per condition (eg, depression, smoking cessation) paired with terms such as “free online” and “self-help.” For each condition, we looked into the organic results found within the first two pages because studies have indicated that a negligible portion of users go beyond the second page [41,42]. If a source referring to a list of programs was found among these organic searches, we included those programs as well. The lists created through the searches for mobile apps and websites were then screened by title to remove duplicates and exclude irrelevant programs (eg, magazine). Programs with unclear titles were examined (by a person who was not one of the program quality raters) using Google Play or website home page prior to exclusion. Using a randomization website [43], 24 eHealth programs were then randomly selected for each of the four conditions—two delivery mediums (mobile/website) × two clinical aims (health-related behavior/mental health)—reaching a total of 96 programs. For example, during this process 24 mobile apps that targeted health-related behavior were randomly selected.

#### Raters’ Training

Different raters evaluated the quality (KF) and checklist (NM) sections of the programs, with the study’s leading author (AB) acting as the second independent rater for both sections. A total of 12 programs (the first three from the randomized lists for each of the four conditions) were used to pilot-test the scale. As part of this process, we also examined programs from other domains that were recommended by experts for their very high quality. This approach enabled coders to locate transcription errors and refine the coding scheme. One goal of the development was to achieve high interrater reliability at the construct level so that Enlight users could reliably present and compare construct scores between different programs. To achieve this goal, the refinement process followed the methods for developing benchmarks in thematic and projection test development within the psychology domain [44-46]. During this process, benchmarks were written, codes were refined, and a preliminary manual was developed. Once the raters had rated the programs (independently), ratings were shared simultaneously. Raters then met to discuss the ratings, and to make proper clarifications and adjustments of the scale benchmarks. To minimize potential biases, when disagreement occurred, a third person examined the blinded ratings and further discussed the ratings to enable the final refinement of codes.

#### Reliability Testing

In line with a previous examination in this field [11], a minimum sample size of 41 was required to establish with 87% assurance whether true interrater reliability lay within 0.15 of a sample observation of 0.80 [47,48]. Therefore, we rated 42 mobile apps (21 targeting mental health and 21 targeting health-related behaviors) and 42 website programs (with the same distribution). Accordingly, our reliability testing also included 42 programs targeting mental health and 42 programs targeting health-related behaviors, for a total of 84 eHealth intervention programs that were independently rated.

This paper presents the analysis of all Enlight categories based on raters’ ratings, except for security checklist because security items are based on information retrieved from parties with access to products’ servers (and not on raters’ ratings). The interrater reliability for each of the quality assessment subscales was measured using an intraclass correlation coefficient (ICC) [49] utilizing the two-way mixed effects model with absolute agreement [50]. The internal consistency of the core domains were calculated using Cronbach alpha, which reflects how closely related a set of items are as a group [51]. Internal consistency was not examined for items related to objective requirements on a categorical scale (Enlight checklists) because homogeneity among the checklist items was not assumed (different checklist items might relate to different parts of the construct) [52]. Therefore, interrater reliability for each categorical item was determined. Cohen kappa, which measures the achieved agreement between two raters above and beyond the overall probability of random agreement, was applied [53].

#### Further Analysis

Overall, differences between delivery mediums and clinical aims in terms of quality scores and intercorrelations were examined while adjusting *P* values based on the Benjamini-Hochberg correction [54]. The correlation matrix between different quality assessments was examined using Pearson correlations. The relationship between these assessments was also examined using a conditional probability approach, which measures the probability of an event given that another event has occurred [55]. This analysis aimed to examine the percentage of products that met a certain quality standard from among a total sample of products that met another quality standard. This method enabled us to examine whether a certain range scores in one quality construct were associated with a similar range of scores in a different construct.

Preliminary concurrent validity was assessed by examining the correlations between different quality constructs and selected variables that were expected to relate either to a program’s acceptability or its efficacy. Combined quality construct scores based on means of several quality constructs were also added to this analysis to examine the benefits of summarizing several concepts into a single score. Two of the selected variables, credibility checklist and programs backed by research evidence (evidence-based program), were developed as part of Enlight and will be described in the Results section related to Enlight development.

The third variable, program popularity, aimed to examine preliminary acceptability based on the number of people choosing to use it. For websites, we recorded the Alexa traffic rank [56], which estimates a website’s popularity based on a combination of mean daily visitors and page views [57]. This traffic rank was used following a preliminary step in which we compared ranks to the SimilarWeb traffic estimator [58], obtaining similar results. We excluded Web-based programs nested within larger websites from the analysis because a high percentage of users were expected to access the website for reasons other than the intervention program. The number of mobile app downloads was taken from Google Play, which presents the range of downloads (eg, 500-1000) for each app; for each program, the lower limit was documented (eg, 500).

## Results

### Enlight Development

The electronic and manual searches produced a total of 7903 records (see Multimedia Appendix 4 for a flow diagram). Through the first screening process, 181 papers were identified and retrieved for detailed evaluation and a total of 99 sources met all inclusion criteria (76 papers from peer-reviewed journals, 9 papers from conference proceedings, 7 manuscripts, 6 websites, and 1 book). A complete list of sources used in the criteria-gathering process is available with this paper (see Multimedia Appendix 5, sources list).

Overall, 1252 items were extracted from the sources; 143 were found to be not relevant for the evaluation of eHealth products and 633 were deemed to be duplicates, leaving a total of 476 criteria. Identified criteria were then grouped and organized in an iterative process into 10 constructs (see Table 1) and three sections to create Enlight: Classification (ie, classifying the program based on acknowledged categories), quality assessment, and checklists (Multimedia Appendix 6, Enlight).

**Table 1:** Frequency of explicit evaluation criteria for eHealth interventions by different constructs (N=476).

Criteria constructs		n (%)
Classification (intended users, clinical condition, program aim)		19 (4.0)
Usability (navigation, learnability, ease of use)		48 (10.1)
Visual design (aesthetics, layout, size)		35 (7.4)
User engagement (content presentation, interactive, not irritating, targeted/tailored/personalized, captivating)		45 (9.5)
Content (evidence-based content, quality of information provision, complete and concise, clarity about program’s purpose)		79 (16.6)
Therapeutic persuasiveness (call for action, load reduction of activities, therapeutic rationale and pathway, rewards, real data driven/adaptive, ongoing feedback, expectations and relevance)		92 (19.3)
Therapeutic alliance (basic acceptance and support, positive therapeutic expectations, relatability)		45 (9.5)
General subjective evaluation (appropriate features to meet clinical aim, right mix of ability and motivation, likability)		36 (7.6)
Credibility^a^ (owner’s credibility, maintenance, strong advisory support, third-party endorsement, evidence for successful implementation, evidence-based program)		49 (10.3)
Privacy and security (terms of use, information on social platforms, security of data and transmission, documentation of data exposure, compliance, third-party endorsement)		28 (5.9)

^a^ Also includes evidence-based program that is ranked and examined separately.

#### Quality Assessment Section

The quality assessment section was designed to capture the different qualities of eHealth interventions. It consists of 25 items divided into six core constructs related to the eHealth intervention program: usability, visual design, user engagement, content, therapeutic persuasiveness, and therapeutic alliance. Another construct, general subjective evaluation (of program’s potential), asks the rater to subjectively evaluate the program as a whole following the completion of the core concept ratings. All constructs were based on heuristic evaluation to enable the examination of programs independently of empirical examination, built on a scale of 1 to 5 (1=very poor; 2=poor; 3=fair; 4=good; 5=very good), and calculated by averaging the items of which they are comprised.

#### Checklists Section

Overall, the checklists were based on acknowledged criteria that cover distinct domains related to product use and include credibility, evidence-based program (as a distinct part of programs’ credibility), privacy explanation, and basic security. These checklists are not expected to directly impact the end user’s experience of the product’s efficacy; however, the criteria contained in the lists may expose the user (or provider) to acknowledged risks or benefits. These measures are calculated by aggregating the scores received in each of the respective categorical items—excluding evidence-based program because it is based on a five-point scale. The privacy explanation and basic security checklists are the only measures in which a lower score equates to better quality. The basic security checklist is the only measure that is not based on a rater’s rating, but rather on information retrieved from parties with access to the product’s servers.

### Enlight Testing

#### Reliability Testing

The electronic searches produced a total of 2227 mobile apps and 1283 Web-based programs (see Multimedia Appendix 4 for a flow diagram). Through the first screening process, 235 apps and 502 websites were excluded as duplicates and 1509 apps and 665 websites were excluded for not meeting the inclusion criteria. This left a total of 523 apps and 116 Web-based programs. From these, 96 eHealth programs were chosen through the randomization procedure, 12 of which were used for the training process and 84 for the reliability examination. The scores received by the various programs used for this paper’s analyses are available in Multimedia Appendix 7 (programs’ scores).

Tables 2 and 3 present the descriptive statistics, Cronbach alphas, and ICCs of the categories analyzed by Enlight. The descriptive statistics of the items constituting the quality assessment section are available in Multimedia Appendix 8 (Descriptive statistics of quality section items) and the interrater kappa reliability scores for the credibility checklist and privacy explanation checklist items (which were in the substantial to outstanding agreement range) are available in Multimedia Appendix 9 (kappa reliability scores of credibility and privacy explanation checklists items).

**Table 2:** Descriptive statistics, Cronbach alphas (α), and intraclass correlations (ICC) of assessment scores by different delivery mediums.

Clinical aim	Total (N=84)			Mobile (n=42)			Website (n=42)		
Quality ratings	Mean (SD)	α	ICC (95% CI)	Mean (SD)	α	ICC (95% CI)	Mean (SD)	α	ICC (95% CI)
Usability	3.31 (0.69)	.83	.91 (.86-.94)	3.46 (0.71)	.85	.82 (.68-.91)	3.17 (0.65)	.79	.96 (.92-.98)
Visual design	2.81 (0.82)	.84	.77 (.64-.85)	2.93 (0.83)	.88	.80 (.63-.89)	2.68 (0.79)	.85	.74 (.52-.86)
User engagement	2.62 (0.80)	.88	.90 (.78-.94)	2.47 (0.83)	.91	.92 (.83-.96)	2.78 (0.73)	.85	.85 (.64-.93)
Content	3.00 (0.98)	.90	.93 (.89-.96)	2.40^a^ (0.87)	.91	.91 (.83-.95)	3.59^a^ (0.68)	.78	.85 (.73-.92)
Therapeutic persuasiveness	2.23 (0.68)	.88	.88 (.78-.93)	2.11 (0.71)	.88	.93 (.86-.97)	2.35 (0.62)	.87	.78 (.55-.89)
Therapeutic alliance	2.20 (0.75)	.83	.89 (.72-.95)	1.99 (0.72)	.83	.87 (.72-.94)	2.40 (0.73)	.82	.87 (.54-.95)
General subjective evaluation	2.09 (0.91)	.89	.83 (.73-.89)	1.89 (0.84)	.88	.85 (.73-.92)	2.29 (0.93)	.89	.73 (.50-.86)
Credibility checklist	3.14 (1.50)	—^b^	.95 (.92-.97)	2.21^a^ (1.16)	—	.95 (.90-.97)	4.07^a^ (1.20)	—	.95 (.90-.97)
Evidence-based program	1.32 (0.66)	—^c^	.94 (.91-.96)	1.07^a^ (0.34)	—	.92 (.86-.96)	1.57^a^ (0.80)	—	.94 (.88-.97)
Privacy explanation checklist	2.76 (1.58)	—^b^	.98 (.97-.99)	3.33^a^ (1.26)	—	.99 (.98-.99)	2.19^a^ (1.67)	—	.97 (.95-.98)

^a^ The groups (within the construct) differed significantly at Benjamini-Hochberg adjusted *P*<.05 in *t* test for two independent samples.

^b^ Measure of agreement per categorical item (kappa) is presented in Multimedia Appendix 7.

^c^ The score is based on one item; therefore, Cronbach alpha could not be calculated.

**Table 3:** Descriptive statistics, Cronbach alphas (α), and intraclass correlations (ICC) of assessment scores by different clinical aims.

Clinical aim	Health-related behaviors (n=42)			Mental health (n=42)		
Quality ratings	Mean (SD)	α	ICC (95% CI)	Mean (SD)	α	ICC (95% CI)
Usability	3.29 (0.77)	.83	.92 (.85-.96)	3.34 (0.61)	.84	.88 (.55-.96)
Visual design	2.79 (0.78)	.84	.78 (.55-.89)	2.82 (0.87)	.84	.79 (.55-.93)
User engagement	2.64 (0.79)	.90	.95 (.91-.97)	2.60 (0.81)	.84	.84 (.52-.94)
Content	2.90 (0.93)	.90	.86 (.74-.92)	3.09 (1.03)	.90	.95 (.91-.98)
Therapeutic persuasiveness	2.28 (0.66)	.86	.87 (.76-.93)	2.18 (0.70)	.90	.89 (.62-.95)
Therapeutic alliance	2.03 (0.70)	.77	.73 (.41-.87)	2.37 (0.78)	.87	.89 (.65-.95)
General subjective evaluation	2.03 (0.86)	.88	.72 (.48-.85)	2.15 (0.95)	.89	.85 (.73-.92)
Credibility checklist	2.88 (1.35)	—	.93 (.87-.97)	3.41 (1.61)	—	.96 (.92-.98)
Evidence-based program	1.21 (0.52)	—	.96 (.92-.98)	1.43 (0.77)	—	.93 (.88-.96)
Privacy explanation checklist	3.14 (1.44)	—	.97 (.94-.98)	2.38 (1.64)	—	.99 (.98-.99)

The internal consistencies of the Enlight categories were very high for the total sample (Cronbach alpha: range .83-.90, median .88) and also when separated into delivery mediums (mobile Cronbach alpha: range .83-.91, median .88; website Cronbach alpha: range .78-.89, median .85) or clinical aims (health-related behaviors Cronbach alpha: range .77-.90, median .86; mental health Cronbach alpha: range .84-.90, median .87). The interrater reliabilities of the Enlight categories were in the excellent to almost perfect agreement range for the total sample (ICC: range .77-.98, median .91) and also when separated into delivery mediums (mobile ICC: range .82-.99, median .92; website ICC: range .73-.97, median .86) or clinical aims (health-related behaviors ICC: range .72-.97, median .90; mental health ICC: range .79-.99, median .89). As can been seen in Table 2, significant differences were found between Web-based and mobile-based programs in terms of content, credibility checklist, evidence-based program, and privacy explanation checklist, all favoring the Web-based programs. No other significant differences in Enlight categories’ scores were found between delivery mediums and clinical aims.

#### Further Analysis

The Pearson correlations between the quality assessment constructs are presented in Table 4. In the total sample, usability did not correlate with the other constructs. All other constructs exhibited significant, moderate to strong, positive correlations (*r*: range .34-.86; all *P* ≤.001). A pattern of strong, positive correlations was found between user engagement, content, therapeutic persuasiveness, and therapeutic alliance (*r*: range .68-.86; all *P*<.001). The correlations between the quality assessment constructs were similar when separated into the two clinical aims (health-related behaviors, mental health). However, some differences were found between delivery mediums. Therefore, Table 4 also presents the correlation matrix by delivery mediums. In the sample of mobile apps, usability did not correlate with the other constructs. In the sample of Web-based programs, weak-to-moderate, positive correlations were found between usability and the other constructs. Compared to the mobile app sample, the correlations between visual design and most of the other constructs in the Web-based program sample were numerically lower.

**Table 4:** Pearson correlations between quality assessment core concepts in the total sample and by delivery mediums and clinical aims.

Quality ratings		Usability		Visual design		Content		User engagement		Therapeutic persuasiveness	
		*r*	*P*	*r*	*P*	*r*	*P*	*r*	*P*	*r*	*P*
**Total (N=84)**											
	Visual design	.36	.001								
	Content	–.02	.84	.34	.001						
	User engagement	.14	.21	.65	<.001	.68	<.001				
	Therapeutic persuasiveness	.13	.23	.60	<.001	.69	<.001	.86	<.001		
	Therapeutic alliance	.15	.16	.53	<.001	.75	<.001	.73	<.001	.72	<.001
**Mobile (n=42)**												
	Visual design	.16		.31									
	Content	–.05^a^	.76	.74	<.001						
	User engagement	–.03	.87	.72	<.001	.83	<.001				
	Therapeutic persuasiveness	.05^a^	.76	.73	<.001	.85	<.001	.89	<.001		
	Therapeutic alliance	.002^a^	.99	.70	<.001	.81	<.001	.70	<.001	.73	<.001
**Website (n=42)**												
	Visual design	.54	<.001								
	Content	.41^a^	.008	.31	.04						
	User engagement	.46^a^	.002	.67	<.001	.55	<.001				
	Therapeutic persuasiveness	.34	.03	.53	<.001	.60	<.001	.81	<.001		
	Therapeutic alliance	.47^a^	>.002	.51	.001	.73	<.001	.73	<.001	.70	<.001

^a^ Significant differences in Pearson correlation values were found between the delivery mediums (mobile, website) using Fisher Z-transformation at Benjamini-Hoffman adjusted *P*<.05.

To further examine the relationship between usability and the other constructs in the mobile app sample, these correlations were recalculated after excluding mobile apps with very few features (n=12; see mobile apps marked with “a” in studies in Multimedia Appendix 5). These mobile apps were identified by the raters to receive high usability scores only because they were very lean and therefore easy to learn and use, and not because of specific design aspects enhancing their usability. For the remaining sample of mobile apps (n=30), moderate positive correlations were found between usability and the other constructs (visual design: *r*=.55, *P*=.002; user engagement: *r*=.43, *P*=.02; content: *r*=.41, *P*=.03; therapeutic persuasiveness: *r*=.35, *P*=.055; therapeutic alliance, *r*=.57, *P*=.001).

**Figure 1:**
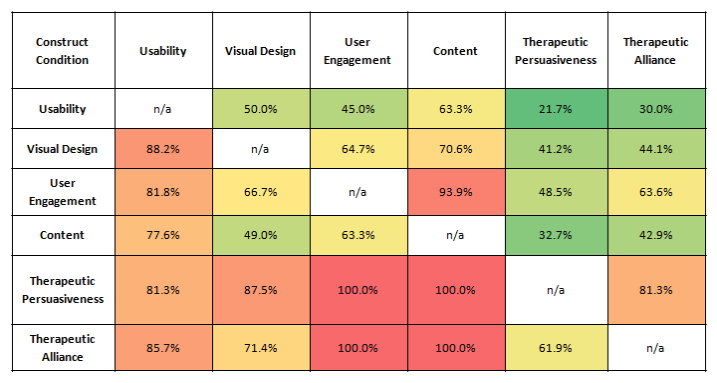
Percentages of eHealth intervention programs with a fair or above score (≥3.0) in quality constructs (columns) out of the sample of programs that received a score of fair or above (≥3.0) in another construct (rows). Within this study sample, higher percentages indicate that having the examined range of scores in one construct (row) improves the chances of receiving the same range of scores in the other construct (column). Fields are colored from higher to lower percentages by the following order: red (highest), orange, yellow, and green (lowest).

To further examine the pattern of strong correlations found between user engagement, content, therapeutic persuasiveness, and therapeutic alliance independent of delivery mediums or clinical aims, a conditional probability analysis was performed by examining the percentage of programs with a score of fair or above (≥3.0) in one construct out of the sample of programs that received a score of fair or above in another construct (Figure 1). Usability and visual design were also added to Figure 1 to present the readers with an overview of all quality constructs.

As Figure 1 shows, 100% of the eHealth intervention programs that received a score of fair or above in therapeutic persuasiveness or therapeutic alliance also received this range of scores in user engagement and content. For programs receiving a score of fair or above in user engagement or content, the percentages of programs receiving the same range of scores in therapeutic persuasiveness or therapeutic alliance ranged between 33% and 64%. In effect, having a fair score in user engagement or content did not necessarily mean that the program also received a fair score in therapeutic persuasiveness or therapeutic alliance. A similar pattern appeared between user engagement and content, where having a fair or above score in user engagement meant that the program most likely had a fair or above score in content (94%), but this pattern was not apparent in the opposite direction. Finally, the figure indicates that most programs that received a score of fair or above in any construct other than usability also received the same range of scores in usability (77.6% to 88.2%).

#### Preliminary Concurrent Validity

Table 5 presents the Pearson correlations between the quality constructs and general subjective evaluation, credibility checklist, evidence-based program (empirical research evidence), and program popularity scores. It is important to note that general subjective evaluation was placed in this table because of its applicability to a general examination of programs and from an organizational point of view; however, this variable is considered to be biased because this evaluation was done following the rater’s full examination of the quality constructs. Three aggregated quality construct scores were also added to this analysis: (1) the mean of user engagement, content, therapeutic persuasiveness, and therapeutic alliance scores; (2) the mean of all quality constructs excluding therapeutic persuasiveness and therapeutic alliance (traditional total), and (3) the mean of all quality constructs (total).

**Table 5:** Pearson correlations between Enlight quality constructs and selected variables.

Quality ratings	General subjective evaluation (n=84)		Credibility checklist (n=84)		Evidence-based program (n=84)		Program popularity (n=70)^a^	
	*r*	*P*	*r*	*P*	*r*	*P*	*r*	*P*
Usability	.18	.11	–.18	.10	.01	.96	.07	.54
Visual design	.57	<.001	–.02	.88	–.06	.58	.27	.02
User engagement	.77	<.001	.22	.04	.22	.050	.40	.001
Content	.70	<.001	.60	<.001	.44	<.001	.12	.34
Therapeutic persuasiveness	.76	<.001	.22	.049	.26	.02	.41	<.001
Therapeutic alliance	.81	<.001	.31	.004	.31	.004	.21	.08
User engagement + content + therapeutic persuasiveness + therapeutic alliance	.84	<.001	.40	<.001	.35	.001	.30	.01
Traditional total^b^	.78	<.001	.26	.02	.23	.03	.30	.01
Total^c^	.83	<.001	.28	.009	.27	.01	.32	.007

^a^ The analysis excluded 14 Web-based programs nested within larger websites (see programs marked with a “b” Multimedia Appendix 5) because a high percentage of users were expected to access the website for reasons other than the intervention program.

^b^ Traditional total=mean of all constructs excluding therapeutic persuasiveness and therapeutic alliance.

^c^ Total=mean of all constructs.

Overall, no single construct had a correlation pattern that outperformed the others; however, user engagement, content, therapeutic persuasiveness, and therapeutic alliance showed positive correlations with all variables, most of which were significant (13/16; *r*: range .22-.81; all *P*<.05). The combined scores showed significant, positive, weak-to-moderate correlations with credibility checklist, evidence-based program, and program popularity scores (*r*: range .23-.40; all *P*<.05); compared to the other combined scores, traditional total showed numerically underperformed results. All combined scores showed significant, positive correlations with all selected variables.

No significant differences in Pearson correlations were found between different delivery mediums or clinical aims using Fisher Z-transformation at Benjamini-Hoffman adjusted *P*<.05 with two exceptions: significant differences in the usability and credibility checklist correlation, and usability and evidence-based program correlation were found between delivery mediums. These correlations were negative in mobile-based programs (*r*=–.34 and *r*=–.37, respectively) and positive in Web-based programs (*r*=.27 and *r*=.31, respectively).

## Discussion

Enlight is a comprehensive suite of assessments developed to evaluate the quality of eHealth intervention programs. It was developed following the first systematic review assessing different aspects of both eHealth and mHealth interventions, including persuasive design, behavior change, and therapeutic alliance principles. As part of the quality assessment section, two concepts that relate to a program’s therapeutic potential and did not appear in previous scales (eg, [10,11]) were introduced: therapeutic persuasiveness and therapeutic alliance. To our knowledge, Enlight is also the first suite of heuristic-based quality measures to include separate sections, one for quality aspects that cover the user’s experience and the other for those quality aspects that do not directly alter the user’s experience of the program (eg, team’s credibility is not part of any construct within the quality assessment section). Combined with the high interrater reliability scores at the construct level (ICC: range .77-.98, median .91), these findings suggest that Enlight differs from previous work by enabling stakeholders to objectively examine individual quality constructs; in that way, Enlight is a suite of scales rather than one quality measure.

The results indicate that it is important to examine therapeutic persuasiveness and therapeutic alliance as part of the main quality constructs of eHealth intervention programs. Most importantly, a conditional probability analysis revealed that 100% of the eHealth intervention programs that received a score of fair or above in therapeutic persuasiveness or therapeutic alliance received the same range of scores in user engagement and content. For programs with fair or above scores in user engagement and content, only 33% to 64% of them received the same range of scores in therapeutic persuasiveness or therapeutic alliance. This means that, despite the strong, positive correlations found between the four aforementioned constructs, the relationships between them are more complicated: achieving a certain standard of scores in user engagement or content does not necessarily mean that this standard will be achieved in the therapeutic constructs and therefore justifies separate ratings of these constructs. Second, when examining the correlations between combined scores and variables that were expected to relate to either the program’s acceptability or its efficacy, the combined score that did not include therapeutic persuasiveness or alliance descriptively underperformed other combined scores, but the difference was small. Nevertheless, altogether these preliminary findings are congruent with findings from other studies, showing that persuasive design and behavior change principles are important factors in understanding the potential of eHealth intervention programs [17,19,20]. These findings also correspond with previous studies suggesting that eHealth intervention programs’ facilitation of a therapeutic alliance may play a role in the understanding of these programs’ potential [28-33].

The analyses pointed to significant, positive correlations between the combined quality scores and credibility, evidence-based program, and program popularity scores (*r*: range .23-.41; all *P* ≤.02). At the construct level, user engagement, content, therapeutic persuasiveness, and therapeutic alliance demonstrated a pattern of positive correlations with these variables, although results were not always significant (13/16, *P*<.05). These preliminary findings relate to Enlight’s concurrent validity, but they should be interpreted with caution because two of the selected variables only indirectly relate to program efficacy. Nevertheless, the program popularity score is related to a product’s acceptance and the evidence-based program score is directly related to the availability of sound research evidence on the product’s efficacy. These findings highlight the potential of this tool despite the need for further examination as discussed later.

### Integrating the Quality Assessment Section Scores

Overall, combining quality constructs into aggregated scores by averaging them was introduced by previous scale developers [47] and is the simplest way to integrate the different constructs. As demonstrated, this integration was supported by empirical evidence showing that all combined scores had significant, positive correlations with the selected variables. However, the study results indicate that it might not be beneficial to combine usability with other scores in a straightforward manner. This is because lean programs may contain very limited content and features can be very easy to learn and use. As a result, such programs yield high usability scores, but very low scores in content, engagement, or therapeutic constructs. Our finding is congruent with previous studies suggesting that usability might need to be considered as a barrier to, rather than a facilitator of, effective interventions (eg, [59,60]). It also might be that in different cases different constructs are more important or redundant. For example, Althoff et al showed that the mobile app “Pokemon Go,” which asks users to move between different physical locations to advance in the game, has contributed to an increase in users’ physical activity [61]. Engagement in these kinds of games might equate to beneficial outcomes; therefore, in this kind of case, the therapeutic persuasiveness construct may become redundant. To conclude, more studies are needed to examine the relationships between the different constructs before determining how to integrate them in a way that accurately captures the potential of different interventions.

### Generalizability

The reliability analysis demonstrated a similar range of interrater agreement and internal consistency in the different delivery mediums and clinical aims. Further analysis revealed similar range of correlations between quality assessment scores and scores of acceptability and efficacy in the different groups, suggesting that these quality ratings account for the same phenomena in these groups. This is the result of applying heuristic-based evaluation techniques that target general principles of quality. For example, principles of therapeutic persuasiveness, such as “therapeutic rational,” or principles of content, such as “information provision,” do not distinguish between delivery mediums or clinical aims. In our review process, we also did not identify important principles that relate to the quality of programs cannot be accounted for by a specific delivery medium. These results extend the work of previous criteria-based tools by pointing at the first time to the possibility of objectively rating different eHealth interventions using one tool, regardless of their delivery medium or clinical aim. Our analyses also identified some significant differences between Web-based and mobile-based programs in terms of content, credibility, evidence-based program, and privacy explanation checklist, all favoring Web-based interventions. It is important to note that Web-based interventions have been around for longer; therefore, there have been more opportunities for empirically based revisions and for scholars to become an integral part of the field.

### Limitations

This study has several limitations that should be addressed. Even though correlations between Enlight quality scores and relevant variables related to concurrent validity were introduced, this does not fully demonstrate criterion validity in directly predicting a program’s acceptability or efficacy. This could be examined once sound data about user analytics and outcome reports are available for a set of rated programs. A further limitation is that at this point we cannot suggest a single strategy for combining quality assessment scores or what range of scores would be good enough to create desired outcomes; rather, we suggest presenting several constructs and examining the relationships between them until there is more evidence backing a specific approach to score integration. As discussed, the high reliability demonstrated by Enlight enables to present all scores at the construct level (since they can be regarded as separate objective metrics).

### Future Directions

Several future directions for research and practice were identified. First, the predictive validity of Enlight could be examined by rating different programs for which metrics of acceptance and efficacy are accessible, and investigating whether and which quality scores predict engagement and efficacy. Second, it could be beneficial to develop a model of the various relationships between different constructs once many programs have been rated. Moreover, specifically examining therapeutic alliance and therapeutic persuasiveness could be helpful when trying to assess the additional need in human support to enhance adherence [62] or to provide any other benefits in eHealth interventions. Third, it could be helpful to examine the applicability of training people to use Enlight based on a complete self-help manual that includes a training kit. Finally, it would be beneficial to examine how this kind of tool can support the decision making of health system leaders when adopting new programs. Efforts are underway to conduct such an examination at Northwell Health, New York.

### Conclusions

This paper provides empirical findings that emphasize the importance of examining persuasive design and therapeutic alliance in the context of quality rating. It also demonstrates the applicability of objectively rating different eHealth interventions using one suite of measures, regardless of their delivery mediums or clinical aims, providing that raters are appropriately trained. The high reliability matrix and preliminary concurrent validity indicate the tool’s potential to examine eHealth programs and the multimodal relationships between different aspects of program quality. More research is needed to establish the tool’s validity for predicting the efficacy of eHealth programs.

## Appendix

Multimedia Appendix 1 Complete PsycINFO database search terms used.

Multimedia Appendix 2 Advisory team.

Multimedia Appendix 3 Search terms used to identify free eHealth intervention programs.

Multimedia Appendix 4 Flow diagrams.

Multimedia Appendix 5 Sources list.

Multimedia Appendix 6 Enlight.

Multimedia Appendix 7 eHealth program scores.

Multimedia Appendix 8 Descriptive statistics of quality section items.

Multimedia Appendix 9 Kappa reliability scores for Credibility and Privacy Explanation Checklists items.

## Abbreviations

ICC: intraclass correlation coefficient
PRISMA: Preferred Reporting Items for Systematic Reviews and Meta-Analyses
