# Supplementary material for: Enlight: A Comprehensive Quality and Therapeutic Potential Evaluation Tool for Mobile and Web-Based eHealth Interventions
Source: J Med Internet Res. 2017 Mar 21;19(3):e82. doi: 10.2196/jmir.7270 (PMC5380814; doi:10.2196/jmir.7270)
Supplement: Multimedia Appendix 1 [file jmir_v19i3e82_app1.pdf]

Multimedia Appendix 1 - PsycINFO Search Terms

(time limits were set through the search engine's platform)

General Principles

1. (Standard* OR principle* OR heuristic* OR norm* OR quality OR criteria OR criterion OR framework OR rating*).m_titl.
2. (measure* OR analy*OR evaluat* or assess*).m_titl.
3. (Mobile* or Mhealth* or Smartphone* or app or apps or ehealth* or e-health* or text or messag* or internet* or web* or video* or software* or digital* or media or technology or technologies).m_titl.
4. #1 AND #2 AND #3

Behavior Change/Persuasive Design

1. (standard* OR principle* OR heuristic* OR norm* OR quality OR criteria OR criterion OR framework OR rating* OR measur* or analy* OR evaluat* OR assess*).m_titl.
2. (persuas* or convinc*).m_titl.
3. (behavior change).m_titl.
4. (behavior AND design).m_titl.
5. (persuasive AND design).m_titl.
6. (#1 AND (#2 OR #3)) OR #4 OR #5

Therapeutic Properties

1. (potential OR quality OR standard* OR principle* OR heuristic* OR norm* OR criteria OR criterion OR framework OR rating*).m_titl.
2. (psychotherapy OR psychotherapeutic).m_titl.
3. (therapeutic).mp. [mp=title, abstract, heading word, table of contents, key concepts, original title, tests & measures]
4. (alliance).mp. [mp=title, abstract, heading word, table of contents, key concepts, original title, tests & measures]
5. (questionnaire).mp. [mp=title, abstract, heading word, table of contents, key concepts, original title, tests & measures]
6. (#1 AND #2) OR (#3 AND #4 AND #5)
