# Supplementary material for: Enlight: A Comprehensive Quality and Therapeutic Potential Evaluation Tool for Mobile and Web-Based eHealth Interventions
Source: J Med Internet Res. 2017 Mar 21;19(3):e82. doi: 10.2196/jmir.7270 (PMC5380814; doi:10.2196/jmir.7270)
Supplement: Multimedia Appendix 2 [file jmir_v19i3e82_app2.pdf]

Multimedia Appendix 2 – Advisory Team

The team consisted of: three licensed clinical psychologists – two with extensive experience in the hands-on development and research of eHealth/mHealth products (Amit Baumel; Fred Muench), and one with two years of experience in content development for eHealth programs (Keren Faber); one psychiatrist with more than 30 years of experience leading research studies, including the development of programs that leverage the novel technology of eHealth products [1], and who is also a senior vice-president of behavioral health services in a large health system in the United States (John Kane); three experienced developers who are also directors of user facing profitable products (Ofer Reichmann, Ohad Samet, Amit Shabtay); two graphic and user experience design professionals – the first (Ofer Holtsman) leading user experience methodology and managing a team of user experience specialists and graphic designers in Netcraft (an international agency providing consultation to companies with user facing digital products), and the second being a products user experience architect – the former vp product of Biogaming – who designed YuGo Microsoft Kinect-based physical therapy system which recently received FDA clearance [2] (Eytan Majar).

References

1. Baumel A, Correll CU, Hauser M, et al. Health technology intervention after hospitalization for schizophrenia: Service utilization and user satisfaction. *Psychiatr. Serv.* 2016;67(9):1035-1038. PMID:27247171.
2. FDA clearance - BioGaming's physiotherapy software. http://www.accessdata.fda.gov/scripts/cdrh/cfdocs/cfpmn/pmn.cfm?ID=K151955. Accessed February 10, 2017. Archived by WebCite® at http://www.webcitation.org/6oBIa65Hf.
