# Supplementary material for: Enlight: A Comprehensive Quality and Therapeutic Potential Evaluation Tool for Mobile and Web-Based eHealth Interventions
Source: J Med Internet Res. 2017 Mar 21;19(3):e82. doi: 10.2196/jmir.7270 (PMC5380814; doi:10.2196/jmir.7270)
Supplement: Multimedia Appendix 3 [file jmir_v19i3e82_app3.pdf]

Multimedia Appendix 3 - Search Terms Used to Identify eHealth Intervention Programs

Websites (Google search)

Health Related Behaviors:

1. (free online)
2. (Diet or weight loss) OR (fitness or physical activity) OR (smoking cessation) OR (alcohol consumption or alcohol abuse) OR (healthy behaviors or healthy habits))
3. (program) OR (training) OR (plan)
4. #1 and #2 and #3

Note: searches ran separately based on OR rules in #2 and #3

Mental Health:

1. (free online)
2. (Depression) OR (Anxiety) OR (Well Being) OR (Mental Health)
3. (self help program) OR (treatment) OR (CBT)
4. #1 and #2 and #3

Mobile (Google Play store)

Health Related Behaviors:
(Diet or weight loss) OR (fitness or physical activity) OR (smoking cessation) OR (alcohol consumption or alcohol abuse) OR (healthy behaviors or healthy habits)

Mental Health:
Depression OR Anxiety OR Mental-Health OR Well-being

Note: searches ran separately based on OR rules
