# Supplementary material for: Enlight: A Comprehensive Quality and Therapeutic Potential Evaluation Tool for Mobile and Web-Based eHealth Interventions
Source: J Med Internet Res. 2017 Mar 21;19(3):e82. doi: 10.2196/jmir.7270 (PMC5380814; doi:10.2196/jmir.7270)
Supplement: Multimedia Appendix 4 [file jmir_v19i3e82_app4.pdf]

APPENDIX 4 - Flow Diagrams of the Systematic Search (4a) and Program Selection (4b)

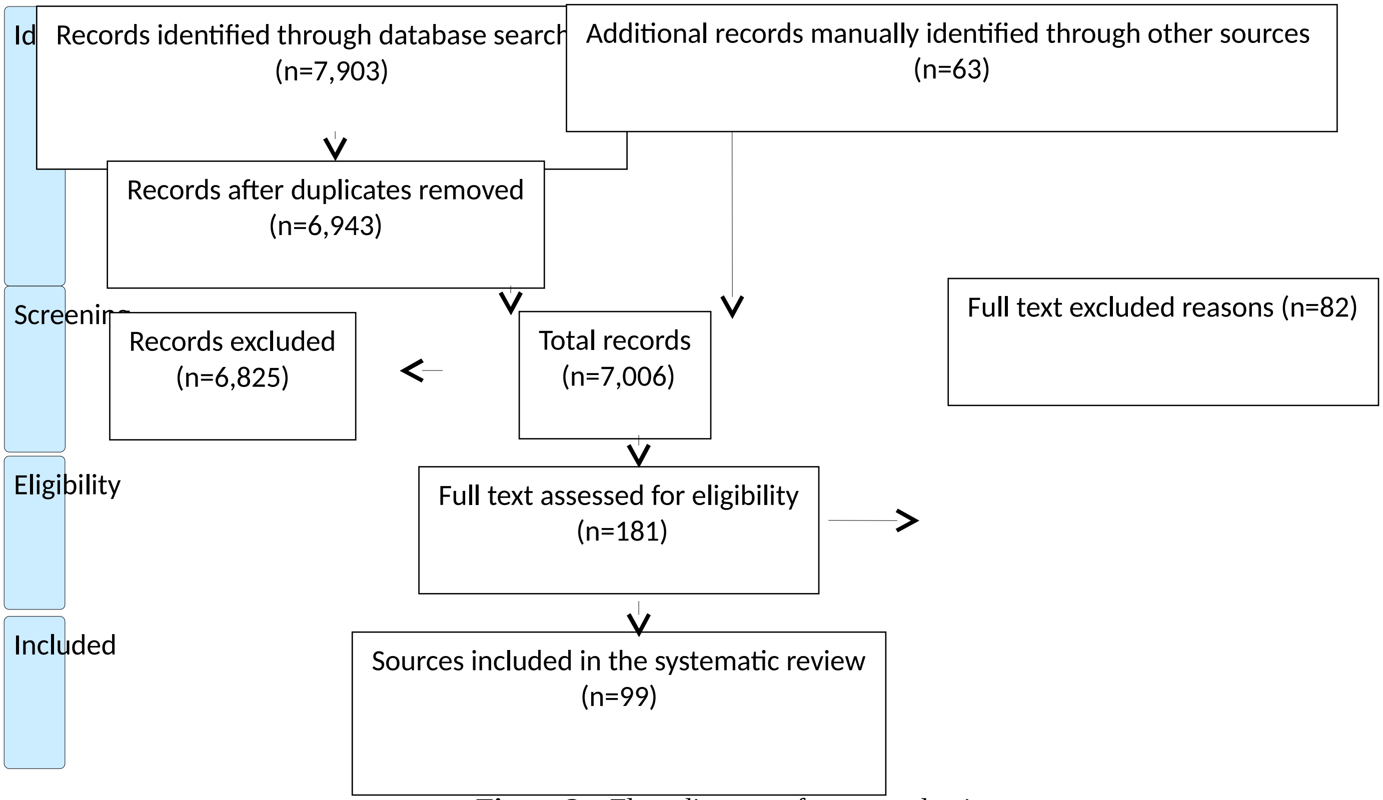

Figure 3a. Flow diagram of source selection

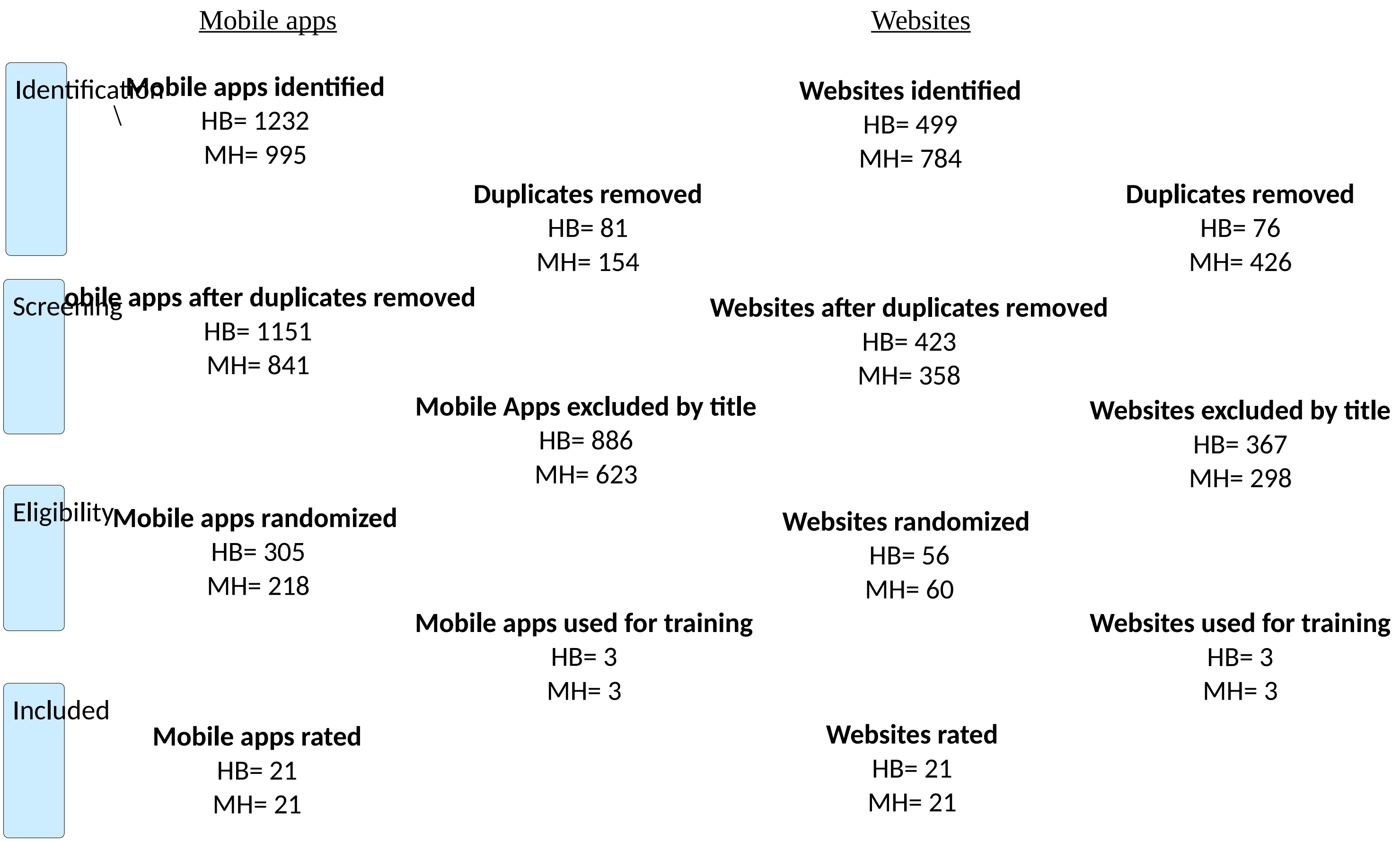

Figure 3b. Flow diagrams of app/website selection
Notes: HB – Health-Related Behaviors; MH – Mental Health
