# Supplementary material for: Enlight: A Comprehensive Quality and Therapeutic Potential Evaluation Tool for Mobile and Web-Based eHealth Interventions
Source: J Med Internet Res. 2017 Mar 21;19(3):e82. doi: 10.2196/jmir.7270 (PMC5380814; doi:10.2196/jmir.7270)
Supplement: Multimedia Appendix 5 [file jmir_v19i3e82_app5.pdf]

Multimedia Appendix 5 – Sources List

General Principles, Behavior change/Persuasive Design

Author/Year	Title	Describes a Scale/ Checklist
**Papers Published in Peer Reviewed Journals**		
Abbott (2000) [1]	Web page quality: can we measure it and what do we find? A report of exploratory findings	Yes
Ademiluyi et al. (2003) [2]	Evaluating the reliability and validity of three tools to assess the quality of health information on the Internet	Yes
Adorisio et al. (2012) [3]	Analysis of readability and quality of web pages addressing both common and uncommon topics in pediatric surgery	Yes
Agarwal et al. (2002) [4]	Assessing a firm's Web presence: A heuristic evaluation procedure for the measurement of usability	Yes
Akter et al. (2013) [5]	Development and validation of an instrument to measure user perceived service quality of mHealth	Yes
Aladwani & Palvia (2002) [6]	Developing and validating an instrument for measuring user-perceived web quality	Yes
Alyusuf et al. (2013) [7]	Development and validation of a tool to evaluate the quality of medical education websites in pathology	Yes
Arora et al. (2014) [8]	Privacy and security in mobile health (mHealth) research	Yes
Baranowski et al. (2010) [9]	Design of video games for children’s diet and physical activity behavior change.	No
Barnes et al. (2003) [10]	Measuring the Relevance of Evaluation Criteria among Health Information Seekers on the Internet	Yes
Baumel & Muench (2016) [11]	Heuristic Evaluation of Ehealth Interventions: Establishing Standards That Relate to the Therapeutic Process Perspective	No
Belmon et al. (2015) [12]	Dutch Young Adults Ratings of Behavior Change Techniques Applied in Mobile Phone Apps to Promote Physical Activity: A Cross-Sectional Survey	Yes
Chatterjee & Price (2009). [13]	Healthy Living with Persuasive Technologies: Framework, Issues, and Challenges	No

Author/Year	Title	Describes a Scale/ Checklist
Cheh et al. (2003) [14]	An assessment of the quality and usability of smoking cessation information on the Internet	No
Chen et al. (2015) [15]	The Most Popular Smartphone Apps for Weight Loss: A Quality Assessment	Yes
Chiu et al. (2014) [16]	Motivating the motivators: Lessons learned from the design and evaluation of a social persuasion system	
Chumber et al. (2015) [17]	A methodology to analyze the quality of health information on the internet: the example of diabetic neuropathy	Yes
Conesa-Fuentes & Hernandez-Morante (2016) [18]	Prospective analysis of the quality of Spanish health information web sites after 3 years	Yes
Conesa-Fuentes et al. (2013) [19]	Evaluation of the quality of the general health information webpages in Spain: influence of page source	Yes
Crane et al. (2015) [20]	Behavior change techniques in popular alcohol reduction apps: content analysis.	Yes
Cristobal et al. (2007) [21]	Perceived e-service quality (PeSQ): Measurement validation and effects on consumer satisfaction and web site loyalty	Yes
Cugelman et al. (2011) [22]	Online interventions for social marketing health behavior change campaigns: a meta-analysis of psychological architectures and adherence factors	Yes
Cummings et al. (2013) [23]	Issues and considerations for healthcare consumers using mobile applications.	No
Cummins et al. (2003) [24]	Development of review criteria to evaluate health behavior change websites	Yes
Curro et al. (2004) [25]	A quality evaluation methodology of health web-pages for non-professionals	Yes
Daraz et al. (2011) [26]	The quality of websites addressing fibromyalgia: an assessment of quality and readability using standardised tools	No
Daud et al. (2013) [27]	An Initial Model of Persuasive Design in Web based Learning Environment	Yes
Davidson (2008) [28]	Six principles of persuasion you can use to influence others	No
Demir & Gozum (2015) [29]	Evaluation of Quality, Content, and Use of the Web Site Prepared for Family Members Giving Care to Stroke Patients	Yes

Author/Year	Title	Describes a Scale/ Checklist
Devine et al. (2016) [30]	Making Quality Health Websites a National Public Health Priority: Toward Quality Standards	Yes
Doshi et al. (2003) [31]	Evaluation of physical activity web sites for use of behavior change theories	Yes
Dragulanescu (2002) [32]	Website Quality Evaluations: Criteria and Tools	Yes
Dubowicz & Schulz (2015) [33]	Medical information on the internet: a tool for measuring consumer perception of quality aspects	Yes
Finstad (2010) [34]	The usability metric for user experience.	Yes
Fogg (1999) [35]	Persuasive Technologies	No
Georgsson et al. (2016) [36]	A Modified User-Oriented Heuristic Evaluation of a Mobile Health System for Diabetes Self-management Support	Yes
Harland & Bath (2007) [37]	Assessing the quality of websites providing information on multiple sclerosis: evaluating tools and comparing sites	Yes
Hsu et al. (2009) [38]	Development of design criteria and evaluation scale for web-based learning platforms	Yes
Idri et al. (2016) [39]	A Framework for Evaluating the Software Product Quality of Pregnancy Monitoring Mobile Personal Health Records	Yes
Irwin et al. (2011) [40]	English and Spanish oral cancer information on the internet: a pilot surface quality and content evaluation of oral cancer web sites	No
Jeon et al. (2014) [41]	Analysis of the information quality of korean obesity-management smartphone applications	No
Kay-Lambkin et al. (2011) [42]	Assessment of function and clinical utility of alcohol and other drug web sites: An observational, qualitative study.	No
Kelders et al. (2012) [43]	Persuasive system design does matter: A systematic review of adherence to web-based interventions	No
Kim et al. (1999) [44]	Published criteria for evaluating health related web sites: Review.	No
Kinzie (2005) [45]	Instructional design strategies for health behavior change	Yes
Lavie & Tractinsky (2004) [46]	Assessing dimensions of perceived visual aesthetics of web sites.	Yes
Martinez-Perez et al. (2013) [47]	Development and evaluation of tools for measuring the quality of experience (QoE) in mHealth applications	Yes
McMillan et al. (2016) [48]	Quality assessment of a sample of mobile app-based health behavior change interventions using a tool based on the National Institute of Health and Care Excellence behavior change guidance	Yes

Author/Year	Title	Describes a Scale/ Checklist
Morera et al. (2016) [49]	Security Recommendations for mHealth Apps: Elaboration of a Developer's Guide	Yes
Morrissey et al. (2016) [50]	Behavior Change Techniques in Apps for Medication Adherence: A Content Analysis.	Yes
Moshagen & Thielsch (2013) [51]	A short version of the visual aesthetics of websites inventory.	Yes
Oinas-Kukkonen & Harjumaa (2009) [52]	Persuasive Systems Design: Key Issues, Process Model and System Features	No
Olsina & Rossi (2002) [53]	Measuring Web application quality with WebQEM	Yes
Provost et al. (2006) [54]	The initial development of the WebMedQual scale: Domain assessment of the construct of quality of health web sites	Yes
Riley et al. (2011) [55]	Health behavior models in the age of mobile interventions: Are our theories up to the task?	No
Shore et al. (2014) [56]	Review of mobile health technology for military mental health.	Yes
Spagnolli et al. (2016) [57]	Interactive persuasive systems: A perspective on theory and evaluation	No
Stoyanov et al. (2015) [58]	Mobile app rating scale: a new tool for assessing the quality of health mobile apps	Yes
Torrente et al. (2013) [59]	Sirius: A heuristic-based framework for measuring web usability adapted to the type of website	Yes
Webb et al. (2010) [60]	Using the internet to promote health behavior change: a systematic review and meta-analysis of the impact of theoretical basis, use of behavior change techniques, and mode of delivery on efficacy	Yes
Wohl et al. (2014) [61]	Building it better: Applying human-computer interaction and persuasive system design principles to a monetary limit tool improves responsible gambling	No
Zhang et al. (2016) [62]	Analysis of the Information Quality of Bariatric Surgery Smartphone Applications Using the Silberg Scale	Yes
**Books and Manuscripts**		
Cialdini (2001) [63]	Harnessing the Science of Persuasion	No

Author/Year	Title	Describes a Scale/ Checklist
Eyal (2014). [64]	Hooked: How to build habit-forming products.	No
Health Care Information Management Systems Society. (2012) [65]	Selecting a mobile app: Evaluating the usability of medical applications	Yes
Monkman & Kushniruk (2013) [66]	A health literacy and usability heuristic evaluation of a mobile consumer health application	No
Naumann & Rolker (2000) [67]	Assessment methods for information quality criteria.	Yes
Nielsen (1995) [68]	Ten usability Heuristics for User Interface Design	No
Singh et al. (2016) [69]	Developing a Framework for Evaluating the Patient Engagement, Quality, and Safety of Mobile Health Applications	No
**<u>Papers published in Conference Proceedings</u>**		
Choe et al. (2013) [70]	Persuasive Performance Feedback: The Effect of Framing on Self-Efficacy	No
Kientz et al. (2010) [71]	Heuristic Evaluation of Persuasive Health Technologies	No
Kuehnhausen & Frost (2013) [72]	Trusting smartphone apps? To install or not to install, that is the question.	Yes
Matsoukas et al. [73]	Expanding DISCERN to create a tool for assessing the quality of Web-based health information resources	Yes
Moustakis et al. (2004) [74]	Website quality assessment criteria.	Yes
Oinas-Kukkonen & Harjumaa (2008) [75]	A Systematic Framework for Designing and Evaluating	No
Schulze & Krömker (2010) [76]	A framework to measure user experience of interactive online products.	No
Seethamraju (2004) [77]	Measurement of user perceived web quality	Yes
Väätäjä et al. (2009) [78]	Developing practical tools for user experience evaluation: a case from mobile news journalism	Yes
**<u>Websites</u>**		
https://blog.kissmetrics.com	A Simple Framework for Building User Engagement Features	No

Author/Year	Title	Describes a Scale/ Checklist
/user-engagement-features-framework [79]		
https://www.adaa.org/finding-help/mobile-apps [80]	ADAA Rating Scale	Yes
http://www.jmir.org/announcement/view/77 [81]	Apps Peer Review	Yes
https://www.nngroup.com/articles/usability-101-introduction-to-usability/ [82]	Usability 101: Introduction to usability	No
http://psyberguide.org/psyberguide-ratings-explanation/ [83]	Psyber Guide	Yes
http://www.usabilitynet.org/home.htm [84]	Usability Sciences	No

Therapeutic Properties/Alliance

Author/Year	Title	Describes a Scale/ Check List
**<u>Papers from Peer Reviewed Journals.</u>**		
Accurso et al. (2013) [85]	Psychometric properties of the Therapeutic Alliance Scale for Caregivers and Parents	Yes
Agnew-Davies et al. (1998) [86]	Alliance structure assessed by the Agnew Relationship Measure (ARM)	Yes
Bedregal et al. (2006) [87]	Preliminary Evaluation of the Validity and Reliability of the Spanish Version of the Therapeutic Alliance with Clinician (TAC) Questionnaire	Yes
Blais (2004) [88]	Development of an inpatient treatment alliance scale	Yes
Blais et al. (2010) [89]	Exploring therapeutic alliance in brief inpatient psychotherapy: A preliminary study	Yes
Cahill et al. (2008) [90]	A review and critical appraisal of measures of therapist-patient interactions in mental health settings.	No
Clarke et al. (2016) [91]	Therapeutic Alliance With a Fully Automated Mobile Phone and Web-Based Intervention: Secondary Analysis of a Randomized Controlled Trial.	No
Duncan et al. (2003) [92]	The Session Rating Scale: Preliminary psychometric properties of a “working” alliance measure.	Yes
Elliott & Wexler (1994) [93]	Measuring the impact of sessions in process€ xperiential therapy of depression: The Session Impacts Scale.	Yes
Hatcher & Barends (1996) [94]	Patients' view of the alliance in psychotherapy: Exploratory factor analysis of three alliance measures	Yes
Horvath & Greenberg (1989) [95]	Development and validation of the Working Alliance Inventory.	Yes
Luborsky et al. (1996) [96]	The revised Helping Alliance questionnaire (HAq-II): Psychometric properties	Yes
Mander (2015) [97]	The individual therapy process questionnaire: development and validation of a revised measure to evaluate general change mechanisms in psychotherapy	Yes
Misdrahi et al. (2009) [98]	The 4-Point ordinal Alliance Self-report: A self-report questionnaire for assessing therapeutic relationships in routine mental health	Yes

Author/Year	Title	Describes a Scale/ Check List
**Manuscript**		
VanderWal (2002) [99]	Examination of Therapeutic Alliance and Dependent-Care Agency in the Context of Complementary and Alternative Therapy Utilization by Mothers for Their Children with Asthma.	Yes

References - General Principles and Persuasive Design

Peer Reviewed Papers

1. Abbott, V.P. (2000). Web page quality: can we measure it and what do we find? A report of exploratory findings. *Journal of Public Health* 22(2), 191-197.
2. Ademiluyi, G., Rees, C. E., & Sheard, C. E. (2003). Evaluating the reliability and validity of three tools to assess the quality of health information on the Internet. *Patient Education and Counseling*, *50*(2), 151-155.
3. Adorisio, O., Silveri, M., Rivosecchi, M., Tozzi, A. E., Scottoni, F., & Buonuomo, P. S. (2012). Analysis of readability and quality of Web pages addressing both common and uncommon topics in pediatric surgery. *European Journal of Pediatric Surgery*, *22*(3), 228-233.
4. Agarwal, R., & Venkatesh, V. (2002). Assessing a firm's Web presence: a heuristic evaluation procedure for the measurement of usability. *Information Systems Research*, *13*(2), 168-186.
5. Akter, S., D'Ambra, J., & Ray, P. (2013). Development and validation of an instrument to measure user perceived service quality of mHealth. *Information & Management*, *50*(4), 181-195.
6. Aladwani, A. M., & Palvia, P. C. (2002). Developing and validating an instrument for measuring user-perceived web quality. *Information & Management*, *39*(6), 467-476.
7. Alyusuf, R. H., Prasad, K., Satir, A. M. A., Abalkhail, A. A., & Arora, R. K. (2013). Development and validation of a tool to evaluate the quality of medical education websites in pathology. *Journal of Pathology Informatics, 4(29).*
8. Arora, S., Yttri, J., & Nilsen, W. (2014). Privacy and security in mobile health (mHealth) research. *Alcohol Research: Current Reviews*, *36*(1), 143-151.
9. Baranowski, T., Thompson, D., Buday, R., Lu, A. S., & Baranowski, J. (2010). Design of video games for children's diet and physical activity behavior change. *International Journal of Computer Science in Sport, 9*(2), 3-17.
10. Barnes, M. D., Penrod, C., Neiger, B. L., Merrill, R. M., Thackeray, R., Eggett, D. L., & Thomas, E. (2003). Measuring the relevance of evaluation criteria among health information seekers on the Internet. *Journal of Health Psychology*, *8*(1), 71-82.
11. Baumel, A., & Muench, F. (2016). Heuristic evaluation of Ehealth interventions: establishing standards that relate to the therapeutic process perspective. *JMIR mental health*, *3*(1), e5.
12. Belmon, L. S., Middelweerd, A., te Velde, S. J., & Brug, J. (2015). Dutch young adults ratings of behavior change techniques applied in mobile phone apps to promote physical activity: a cross-sectional survey. *JMIR mHealth and uHealth*, *3*(4), e103.
13. Chatterjee, S., & Price, A. (2009). Healthy living with persuasive technologies: framework, issues, and challenges. *Journal of the American Medical Informatics Association*, *16*(2), 171-178.
14. Cheh, J. A., Ribisl, K. M., & Wildemuth, B. M. (2003). An assessment of the quality and usability of smoking cessation information on the Internet. *Health Promotion Practice*, *4*(3), 278-287.
15. Chen, J., Cade, J. E., & Allman-Farinelli, M. (2015). The most popular smartphone apps for weight loss: a quality assessment. *JMIR mHealth and uHealth*, *3*(4), e104.
16. Chiu, M. C., Chen, C. C. H., Chang, S. P., Chu, H. H., Wang, C., Hsiao, F. H., & Huang, P. (2014). Motivating the motivators: lessons learned from the design and evaluation of a social persuasion system. *Pervasive and Mobile Computing, 10*(B), 203-221.

17. Chumber, S., Huber, J., & Ghezzi, P. (2015). A methodology to analyze the quality of health information on the internet: the example of diabetic neuropathy. *The Diabetes Educator*, *41*(1), 95-105.
18. Conesa-Fuentes, M. C., & Hernandez-Morante, J. J. (2016). Prospective analysis of the quality of Spanish health information web sites after 3 years. *Informatics for Health and Social Care*, *41*(4), 417-29..
19. Conesa-Fuentes, M. C., Aguinaga-Ontoso, E., & Hernandez-Morante, J. J. (2013). Evaluation of the quality of the general health information Web-pages in Spain: influence of page source. *Informatics for Health and Social Care*, *38*(4), 382-395.
20. Crane, D., Garnett, C., Brown, J., West, R., & Michie, S. (2015). Behavior change techniques in popular alcohol reduction apps: content analysis. *Journal of Medical Internet Research*, *17*(5), e118.
21. Cristobal, E., Flavián, C., & Guinalíu, M. (2007). Perceived e-Service Quality (PeSQ) measurement validation and effects on consumer satisfaction and web site loyalty. *Managing Service Quality: An International Journal*, *17*(3), 317-340.
22. Cugelman, B., Thelwall, M., & Dawes, P. (2011). Online interventions for social marketing health behavior change campaigns: a meta-analysis of psychological architectures and adherence factors. *Journal of Medical Internet Research*, *13*(1), e17.
23. Cummings E., Borycki E.M., & Roehrer E. (2013). Issues and considerations for healthcare consumers using mobile applications. *Studies in Health Technology and Informatics, 183*, 227-31.
24. Cummins, C. O., Prochaska, J. O., Driskell, M. M., Evers, K. E., Wright, J. A., Prochaska, J. M., & Velicer, W. F. (2003). Development of review criteria to evaluate health behavior change websites. *Journal of Health Psychology*, *8*(1), 55-62.
25. Currò, V., Buonuomo, P. S., Onesimo, R., De Rose, P., Vituzzi, A., Di Tanna, G. L., & D'atri, A. (2004). A quality evaluation methodology of health Web-pages for non-professionals. *Medical Informatics and the Internet in Medicine*, *29*(2), 95-107.
26. Daraz, L., MacDermid, J. C., Wilkins, S., Gibson, J., & Shaw, L. (2011). The quality of websites addressing fibromyalgia: an assessment of quality and readability using standardised tools. *BMJ Open*, *1*(1), e000152.
27. Daud, N. A., Sahari, N., & Muda, Z. (2013). An initial model of persuasive design in web based learning environment. *Procedia Technology*, *11*, 895-902.
28. Davidson, T. W. (2008). Six principles of persuasion you can use to influence others. *Physician Executive*, *34*(5), 20-23.
29. Demir, Y., & Gozum, S. (2015). Evaluation of quality, content, and use of the Web site prepared for family members giving care to stroke patients. *Computers, Informatics, Nursing*, *33*(9), 396-403.
30. Devine, T., Broderick, J., Harris, L. M., Wu, H., & Hilfiker, S. W. (2016). Making quality health websites a national public health priority: toward quality standards. *Journal of Medical Internet Research*, *18*(8), e211.
31. Doshi, A., Patrick, K., Sallis, J. F., & Calfas, K. (2003). Evaluation of physical activity web sites for use of behavior change theories. *Annals of Behavioral Medicine*, *25*(2), 105-111.
32. Dragulanescu, N. G. (2002). Website quality evaluations: criteria and tools. *The International Information & Library Review*, *34*(3), 247-254.
33. Dubowicz, A., & Schulz, P. J. (2015). Medical information on the internet: a tool for measuring consumer perception of quality aspects. *Interactive Journal of Medical Research*, *4*(1), e8.

34. Finstad, K. (2010). The usability metric for user experience. *Interacting with Computers*, *22*(5), 323-327.
35. Fogg, B. J. (1999). Persuasive technologies. *Communications of the ACM*, *42*(5), 27-29.
36. Georgsson, M., Staggers, N., & Weir, C. (2016). A modified user-oriented heuristic evaluation of a mobile health system for diabetes self-management support. *Computers, Informatics, Nursing*, *34*(2), 77-84.
37. Harland, J., & Bath, P. (2007). Assessing the quality of websites providing information on multiple sclerosis: evaluating tools and comparing sites. *Health Informatics Journal*, *13*(3), 207-221.
38. Hsu, C. M., Yeh, Y. C., & Yen, J. (2009). Development of design criteria and evaluation scale for web-based learning platforms. *International Journal of Industrial Ergonomics*, *39*(1), 90-95.
39. Idri, A., Bachiri, M., & Fernández-Alemán, J. L. (2016). A framework for evaluating the software product quality of pregnancy monitoring mobile personal health records. *Journal of Medical Systems*, *40*(3), 1-17.
40. Irwin, J. Y., Thyvalikakath, T., Spallek, H., Wali, T., Kerr, A. R., & Schleyer, T. (2011). English and Spanish oral cancer information on the Internet: a pilot surface quality and content evaluation of oral cancer Web sites. *Journal of Public Health Dentistry*, *71*(2), 106-116.
41. Jeon, E., Park, H., Min, Y. H., & Kim, H. Y. (2014). Analysis of the information quality of Korean obesity-management smartphone applications. *Healthcare Informatics Research*, *20*(1), 23-29.
42. Kay-Lambkin, F. J., White, A., Baker, A. L., Kavanagh, D. J., Klein, B., Proudfoot, J., Drennan, J., Connor, J.,& Young, R. M. (2011). Assessment of function and clinical utility of alcohol and other drug web sites: an observational, qualitative study. *BMC Public Health*, *11*(1), 277.
43. Kelders, S. M., Kok, R. N., Ossebaard, H. C., & Van Gemert-Pijnen, J. E. (2012). Persuasive system design does matter: a systematic review of adherence to web-based interventions. *Journal of Medical Internet Research*, *14*(6), e152.
44. Kim, P., Eng, T. R., Deering, M. J., & Maxfield, A. (1999). Published criteria for evaluating health related web sites: review. *BMJ*, *318*(7184), 647-649.
45. Kinzie, M. B. (2005). Instructional design strategies for health behavior change. *Patient Education and Counseling*, *56*(1), 3-15.
46. Lavie, T., & Tractinsky, N. (2004). Assessing dimensions of perceived visual aesthetics of web sites. *International Journal of Human-Computer Studies*, *60*(3), 269-298.
47. Martínez-Pérez, B., de la Torre-Díez, I., Candelas-Plasencia, S., & López-Coronado, M. (2013). Development and evaluation of tools for measuring the Quality of Experience (QoE) in mHealth applications. *Journal of Medical Systems*, *37*(5), 1-8.
48. McMillan, B., Hickey, E., Patel, M. G., & Mitchell, C. (2016). Quality assessment of a sample of mobile app-based health behavior change interventions using a tool based on the National Institute of Health and Care Excellence behavior change guidance. *Patient Education and Counseling*, *99*(3), 429-435.
49. Morera, E. P., de la Torre Díez, I., Garcia-Zapirain, B., López-Coronado, M., & Arambarri, J. (2016). Security recommendations for mHealth apps: elaboration of a developer's guide. *Journal of Medical Systems*, *40*(6), 1-13.
50. Morrissey, E. C., Corbett, T. K., Walsh, J. C., & Molloy, G. J. (2016). Behavior change techniques in apps for medication adherence: a content analysis. *American Journal of Preventive Medicine*, *50*(5), e143-e146.

51. Moshagen, M., & Thielsch, M. (2013). A short version of the visual aesthetics of websites inventory. *Behaviour & Information Technology*, *32*(12), 1305-1311.
52. Oinas-Kukkonen, H., & Harjumaa, M. (2009). Persuasive systems design: Key issues, process model, and system features. *Communications of the Association for Information Systems*, *24*(1), 485-501.
53. Olsina, L., & Rossi, G. (2002). Measuring Web application quality with WebQEM. *IEEE Multimedia*, *9*(4), 20-29.
54. Provost, M., Koompalum, D., Dong, D., & Martin, B. C. (2006). The initial development of the WebMedQual scale: domain assessment of the construct of quality of health web sites. *International Journal of Medical Informatics*, *75*(1), 42-57.
55. Riley, W. T., Rivera, D. E., Atienza, A. A., Nilsen, W., Allison, S. M., & Mermelstein, R. (2011). Health behavior models in the age of mobile interventions: are our theories up to the task? *Translational Behavioral Medicine*, *1*(1), 53-71.
56. Shore, J. H., Aldag, M., McVeigh, F. L., Hoover, R. L., Ciulla, R., & Fisher, A. (2014). Review of mobile health technology for military mental health. *Military Medicine*, *179*(8), 865-878.
57. Spagnolli, A., Chittaro, L., & Gamberini, L. (2016). Interactive persuasive systems: A perspective on theory and evaluation. *International Journal of Human-Computer Interaction*, *32*(3), 177-189.
58. Stoyanov, S. R., Hides, L., Kavanagh, D. J., Zelenko, O., Tjondronegoro, D., & Mani, M. (2015). Mobile app rating scale: a new tool for assessing the quality of health mobile apps. *JMIR mHealth and uHealth*, *3*(1), e27.
59. Torrente, M. C. S., Prieto, A. B. M., GutiéRrez, D. A., & De Sagastegui, M. E. A. (2013). Sirius: a heuristic-based framework for measuring Web usability adapted to the type of website. *Journal of Systems and Software*, *86*(3), 649-663.
60. Webb, T., Joseph, J., Yardley, L., & Michie, S. (2010). Using the internet to promote health behavior change: a systematic review and meta-analysis of the impact of theoretical basis, use of behavior change techniques, and mode of delivery on efficacy. *Journal of Medical Internet Research*, *12*(1), e4.
61. Wohl, M. J., Parush, A., Kim, H. A. S., & Warren, K. (2014). Building it better: applying human–computer interaction and persuasive system design principles to a monetary limit tool improves responsible gambling. *Computers in Human Behavior*, *37*, 124-132.
62. Zhang, M. W., Ho, R. C., Hawa, R., & Sockalingam, S. (2016). Analysis of the information quality of bariatric surgery smartphone applications using the Silberg scale. *Obesity surgery*, *26*(1), 163-168.
63. Cialdini, R. B. (2001). Harnessing the science of persuasion. *Harvard Business Review*, *79*(9), 72-81.
64. Eyal, N. (2014). *Hooked: How to build habit-forming products*. Penguin: Canada.
65. Health Care Information Management Systems Society (2012). *Selecting a mobile app: evaluating the usability of medical applications*. mHIMSS App Usability Work Group: United States.
66. Monkman, H., & Kushniruk, A. (2013, August). A health literacy and usability heuristic evaluation of a mobile consumer health application. In *MedInfo* (pp. 724-728).
67. Naumann, F., & Rolker, C. (2000). Assessment methods for information quality criteria. Retrieved from http://edoc.hu-berlin.de/series/informatik-berichte/138/PDF/138.pdf
68. Nielsen, J. (1995). 10 usability heuristics for user interface design. Retrieved from https://www.nngroup.com/articles/ten-usability-heuristics

69. Singh, K., Drouin, K., Newmark, L. P., Rozenblum, R., Lee, J., Landman, A., ... & Bates, D. W. (2016). Developing a Framework for Evaluating the Patient Engagement, Quality, and Safety of Mobile Health Applications. *Issue brief (Commonwealth Fund)*, *5*, 1-11.
70. Choe, E. K., Lee, B., Munson, S., Pratt, W., & Kientz, J. A. (2013). Persuasive performance feedback: The effect of framing on self-efficacy. In *AMIA Annual Symposium Proceedings* (Vol. 2013, p. 825). American Medical Informatics Association.
71. Kientz, J. A., Choe, E. K., Birch, B., Maharaj, R., Fonville, A., Glasson, C., & Mundt, J. (2010, November). Heuristic evaluation of persuasive health technologies. In *Proceedings of the 1st ACM International Health Informatics Symposium* (pp. 555-564). ACM.
72. Kuehnhausen, M., & Frost, V. S. (2013, February). Trusting smartphone apps? To install or not to install, that is the question. In *2013 IEEE International Multi-Disciplinary Conference on Cognitive Methods in Situation Awareness and Decision Support (CogSIMA)* (pp. 30-37). IEEE.
73. Matsoukas, K., Hyun, S., Currie, L., Joyce, M. P., Oliver, J., Patel, S., ... & Bakken, S. (2007, December). Expanding DISCERN to create a tool for assessing the quality of Web-based health information resources. In *AMIA Annual Symposium Proceedings* (pp. 1048-1048). American Medical Informatics Association.
74. Moustakis, V., Litos, C., Dalivigas, A., & Tsironis, L. (2004, November). Website Quality Assessment Criteria. In *Proceedings of the Ninth International Conference on Information Quality* (pp. 59-73).
75. Oinas-Kukkonen, H., & Harjumaa, M. (2008, June). A systematic framework for designing and evaluating persuasive systems. In *International Conference on Persuasive Technology* (pp. 164-176).
76. Schulze, K., & Krömker, H. (2010, August). A framework to measure user experience of interactive online products. In *Proceedings of the 7th International Conference on Methods and Techniques in Behavioral Research* (p. 14). ACM.
77. Seethamraju, R. (2004). Measurement of user perceived web quality. In *ECIS 2004 Proceedings* (p. 176).
78. Väätäjä, H., Koponen, T., & Roto, V. (2009, September). Developing practical tools for user experience evaluation: a case from mobile news journalism. In *European Conference on Cognitive Ergonomics: Designing beyond the Product---Understanding Activity and User Experience in Ubiquitous Environments* (p. 23). VTT Technical Research Centre of Finland.
79. A simple framework for building user engagement features. In Kissmetrics Blog. https://blog.kissmetrics.com/user-engagement-features-framework/. Accessed November 1, 2016. Archived by WebCite® at http://www.webcitation.org/6lhtxDU4N.
80. ADAA Mental Health Apps rates webpage. https://www.adaa.org/finding-help/mobile-apps. Accessed November 1, 2016. Archived by WebCite® at http://www.webcitation.org/6lhtKNvkc.
81. JMIR Publications. http://www.jmir.org/announcement/view/77. Accessed August 10, 2015. Archived by WebCite® at http://www.webcitation.org/6agJfjxOb.
82. Nielsen Norman Group Usability 101 webpage. https://www.nngroup.com/articles/usability-101-introduction-to-usability/. Accessed November 1, 2016. Archived by WebCite® at http://www.webcitation.org/6lhtiZcac.
83. PsyberGuide website. http://psyberguide.org/. Accessed April 10, 2015. Archived by WebCite® at http://www.webcitation.org/6Xh99eQmZ. http://psyberguide.org/.
84. Usability Net website. http://www.usabilitynet.org/home.htm. Accessed November 1, 2016. Archived by WebCite® at http://www.webcitation.org/6lht4Ht3W

References- Therapeutic Alliance

85. Accurso, E. C., Hawley, K. M., & Garland, A. F. (2013). Psychometric properties of the Therapeutic Alliance Scale for Caregivers and Parents. *Psychological Assessment*, *25*(1), 244-252.
86. Agnew-Davies, R., Stiles, W. B., Hardy, G. E., Barkham, M., & Shapiro, D. A. (1998). Alliance structure assessed by the Agnew Relationship Measure (ARM). *British Journal of Clinical Psychology*, *37*(2), 155-172.
87. Bedregal, L. E., Paris Jr, M., Añez, L. M., Shahar, G., & Davidson, L. (2006). Preliminary evaluation of the validity and reliability of the Spanish version of the Therapeutic Alliance with Clinician (TAC) questionnaire. *Social Indicators Research*, *78*(1), 19-32.
88. Blais, M. A. (2004). Development of an inpatient treatment alliance scale. *The Journal of Nervous and Mental Disease*, *192*(7), 487-493.
89. Blais, M. A., Jacobo, M. C., & Smith, S. R. (2010). Exploring therapeutic alliance in brief inpatient psychotherapy: a preliminary study. *Clinical pPsychology & Psychotherapy*, *17*(5), 386-394.
90. Cahil,l J., Barkham, M., Hardy, G., Gilbody, S., Richards, D., Bower, P., Audin K., & Connell, J. (2008).. A review and critical appraisal of measures of therapist–patient interactions in mental health settings. *Health Technolology Assessment*, *12*(24), ix-47.
91. Clarke, J., Proudfoot, J., Whitton, A., Birch, M. R., Boyd, M., Parker, G., Manicavasagar, V., Hadzi-Pavlovic, D.,& Fogarty, A. (2016). Therapeutic alliance with a fully automated mobile phone and Web-based intervention: secondary analysis of a randomized controlled trial. *JMIR Mental Health*, *3*(1), e10.
92. Duncan, B. L., Miller, S. D., Sparks, J. A., Claud, D. A., Reynolds, L. R., Brown, J., & Johnson, L. D. (2003). The Session Rating Scale: preliminary psychometric properties of a "working" alliance measure. *Journal of Brief Therapy*, *3*(1), 3-12.
93. Elliott, R., & Wexler, M. M. (1994). Measuring the impact of sessions in process experiential therapy of depression: the Session Impacts Scale. *Journal of Counseling Psychology*, *41*(2), 166-174.
94. Hatcher, R. L., & Barends, A. W. (1996). Patients' view of the alliance in psychotherapy: exploratory factor analysis of three alliance measures. *Journal of Consulting and Clinical Psychology*, *64*(6), 1326-1336.
95. Horvath, A. O., & Greenberg, L. S. (1989). Development and validation of the Working Alliance Inventory. *Journal of Counseling Psychology*, *36*(2), 223-233.
96. Luborsky, L., Barber, J. P., Siqueland, L., Johnson, S., Najavits, L. M., Frank, A., & Daley, D. (1996). The revised Helping Alliance questionnaire (HAq-II): psychometric properties. *The Journal of Psychotherapy Practice and Research*, *5*(3), 260-271.
97. Mander, J., Schlarb, A., Teufel, M., Keller, F., Hautzinger, M., Zipfel, S., Wittorf, A., & Sammet, I. (2015). The individual therapy process questionnaire: development and validation of a revised measure to evaluate general change mechanisms in psychotherapy. *Clinical Psychology & Psychotherapy*, *22*(4), 328-345.
98. Misdrahi, D., Verdoux, H., Lançon, C., & Bayle, F. (2009). The 4-Point Ordinal Alliance Self-report: a self-report questionnaire for assessing therapeutic relationships in routine mental health. *Comprehensive Psychiatry*,*50*(2), 181-185.
99. VanderWal, M. C. (2002). *Examination of Therapeutic Alliance and Dependent-Care Agency in the Context of Complementary and Alternative Therapy Utilization by Mothers for Their*

Children with Asthma. Master's thesis, Grand Valley State University, Allendale Charter Township, Michigan.
