# Supplementary material for: Enlight: A Comprehensive Quality and Therapeutic Potential Evaluation Tool for Mobile and Web-Based eHealth Interventions
Source: J Med Internet Res. 2017 Mar 21;19(3):e82. doi: 10.2196/jmir.7270 (PMC5380814; doi:10.2196/jmir.7270)
Supplement: Multimedia Appendix 7 [file jmir_v19i3e82_app7.pdf]

# Multimedia Appendix 7 - eHealth Intervention Programs' Scores

| Delivery | Clinical Aim | Name | Usability | Visual Design | UE[C] | Content | TP[C] | TA[C] | General Subjective Evaluation | Credibility | Evidence Based Program | Privacy Checklist |
|---|---|---|---|---|---|---|---|---|---|---|---|---|
| Mobile | Health Related Behaviors | **30-Day Skinny Jeans Challenge** (SparkPeople) | 3.67 | 2.67 | 2.40 | 2.25 | 1.86 | 1.67 | 1.33 | 3.00 | 1.00 | 3.00 |
| Mobile | Health Related Behaviors | **5 Days Weight Loss Plan** (Free Apps for you) [a] | 4.67 | 2.67 | 1.50 | 1.25 | 1.43 | 1.00 | 1.00 | 1.00 | 1.00 | 2.00 |
| Mobile | Health Related Behaviors | **Diet and Weight Loss** (NutriSoft Brazil) [b] | 3.33 | 3.67 | 3.20 | 3.25 | 2.71 | 2.33 | 2.00 | 3.00 | 1.00 | 4.00 |
| Mobile | Health Related Behaviors | **Diet plan weight loss** (Emil Zatodev) | 3.00 | 1.67 | 1.00 | 1.00 | 1.00 | 1.67 | 1.00 | 1.00 | 1.00 | 4.00 |
| Mobile | Health Related Behaviors | **Fast Weight Loss Tips** (Wisefather) [a] | 4.33 | 2.67 | 1.20 | 1.25 | 1.29 | 1.00 | 1.00 | 1.00 | 1.00 | 4.00 |
| Mobile | Health Related Behaviors | **Health Lab by Tictrac** (Tictrac Limited) | 2.67 | 3.67 | 3.80 | 3.75 | 3.43 | 2.33 | 3.33 | 2.00 | 1.00 | 6.00 |
| Mobile | Health Related Behaviors | **Just Move - Fitness Motivation** (WalkOn - Mobile Health Solutions) | 3.33 | 3.00 | 3.20 | 3.00 | 3.14 | 3.00 | 2.33 | 2.00 | 1.00 | 4.00 |
| Mobile | Health Related Behaviors | **LG health** (LG Electronics, Inc.) | 3.67 | 4.00 | 4.20 | 3.25 | 3.29 | 2.33 | 2.67 | 3.00 | 1.00 | 4.00 |

| Delivery | Clinical Aim | Name | Usability | Visual Design | UE[c] | Content | TP[c] | TA[c] | General Subjective Evaluation | Credibility | Evidence Based Program | Privacy Checklist |
|---|---|---|---|---|---|---|---|---|---|---|---|---|
| Mobile | Health Related Behaviors | **Lose weight without dieting** (Harmonic Soft) | 4.00 | 3.67 | 3.80 | 4.00 | 3.29 | 3.33 | 3.33 | 4.00 | 1.00 | 4.00 |
| Mobile | Health Related Behaviors | **My Drink Control** (Berner Gesundheit) [a] | 4.00 | 3.67 | 2.50 | 2.25 | 2.14 | 1.67 | 2.00 | 2.00 | 1.00 | 2.00 |
| Mobile | Health Related Behaviors | **My Trainer Dasi** (Donets Connecting) | 2.67 | 3.33 | 2.80 | 2.50 | 2.00 | 1.67 | 1.00 | 3.00 | 1.00 | 4.00 |
| Mobile | Health Related Behaviors | **myQuitTime - Stop Smoking** (Arete Appware) | 2.67 | 2.00 | 2.60 | 2.00 | 2.29 | 2.00 | 2.00 | 1.00 | 1.00 | 3.00 |
| Mobile | Health Related Behaviors | **One Minute Workout** (w9 software) [a] | 4.33 | 3.00 | 2.75 | 2.75 | 2.57 | 2.33 | 2.33 | 2.00 | 1.00 | 4.00 |
| Mobile | Health Related Behaviors | **Quit Smoking** (Roxo games) [a] | 5.00 | 2.67 | 2.25 | 2.25 | 2.29 | 1.33 | 2.00 | 2.00 | 1.00 | 3.00 |
| Mobile | Health Related Behaviors | **Quit Smoking Cessasion Nation** (Ron Horner) [a] | 3.67 | 2.00 | 2.60 | 2.00 | 2.29 | 1.67 | 1.00 | 2.00 | 1.00 | 3.00 |
| Mobile | Health Related Behaviors | **Reduce Your Drinking** (corporatecom) | 3.00 | 3.00 | 2.50 | 2.25 | 2.00 | 1.33 | 1.00 | 1.00 | 1.00 | 1.00 |
| Mobile | Health Related Behaviors | **Rehapp** (Kirolous Frouk) | 3.67 | 3.00 | 2.50 | 2.00 | 2.00 | 1.33 | 1.33 | 1.00 | 1.00 | 4.00 |
| Mobile | Health Related Behaviors | **Seven - 7 Minute Workout** (Perigee AB) [b] | 3.67 | 4.33 | 3.40 | 3.50 | 3.29 | 3.33 | 4.00 | 3.00 | 1.00 | 3.00 |

| Delivery | Clinical Aim | Name | Usability | Visual Design | UE[c] | Content | TP[c] | TA[c] | General Subjective Evaluation | Credibility | Evidence Based Program | Privacy Checklist |
|---|---|---|---|---|---|---|---|---|---|---|---|---|
| Mobile | Health Related Behaviors | **Sober Time - Sobriety Counter** (SocioSoft) [a] | 3.67 | 2.33 | 3.00 | 2.25 | 1.86 | 1.33 | 2.00 | 3.00 | 1.00 | 6.00 |
| Mobile | Health Related Behaviors | **Stop Smoking In Two Hours** (Juice Master) | 4.33 | 3.00 | 2.20 | 2.25 | 2.00 | 2.67 | 1.67 | 2.00 | 1.00 | 4.00 |
| Mobile | Health Related Behaviors | **Weight Loss for Beginners** (All I Have Is Video Games Studios) | 2.67 | 2.00 | 1.40 | 2.00 | 1.57 | 1.67 | 1.67 | 1.00 | 1.00 | 2.00 |
| Mobile | Mental Health | **Booster Buddy** (Island Health) | 4.00 | 4.67 | 3.50 | 4.00 | 3.29 | 3.33 | 3.67 | 2.00 | 1.00 | 0.00 |
| Mobile | Mental Health | **Break Free Anxiety** (Abele Apps) | 3.00 | 3.00 | 2.80 | 2.00 | 2.14 | 1.67 | 1.00 | 1.00 | 1.00 | 3.00 |
| Mobile | Mental Health | **Breathe2Relax** (T2) | 2.33 | 2.33 | 2.40 | 3.75 | 2.29 | 2.00 | 2.67 | 5.00 | 2.00 | 3.00 |
| Mobile | Mental Health | **Buddhist Meditation Trainer** (Spacebug)[a] | 4.33 | 2.00 | 2.50 | 1.75 | 1.86 | 1.00 | 2.00 | 2.00 | 1.00 | 3.00 |
| Mobile | Mental Health | **Dealing with Depression** (Sun Media Soft) [a] | 4.33 | 3.33 | 1.25 | 1.50 | 1.57 | 1.67 | 1.00 | 1.00 | 1.00 | 2.00 |
| Mobile | Mental Health | **Depression CBT Self-Help Guide** (Excel At Life) | 2.33 | 2.33 | 2.20 | 1.25 | 1.43 | 1.33 | 1.33 | 5.00 | 1.00 | 3.00 |
| Mobile | Mental Health | **Emotion** (LES LABORATOIRES SERVIER) | 3.33 | 3.33 | 3.00 | 3.00 | 2.43 | 2.33 | 2.00 | 1.00 | 1.00 | 1.00 |
| Mobile | Mental Health | **Guide to Depression Self Help** (9to5AppStore) [a] | 4.00 | 1.67 | 1.00 | 1.50 | 1.29 | 1.33 | 1.00 | 1.00 | 1.00 | 4.00 |
| Mobile | Mental Health | **Life Reboot- Fight Depression** (photonapps) | 3.33 | 2.67 | 2.20 | 1.75 | 1.43 | 2.00 | 1.67 | 1.00 | 1.00 | 6.00 |

| Delivery | Clinical Aim | Name | Usability | Visual Design | UE[c] | Content | TP[c] | TA[c] | General Subjective Evaluation | Credibility | Evidence Based Program | Privacy Checklist |
|---|---|---|---|---|---|---|---|---|---|---|---|---|
| Mobile | Mental Health | **MEDITATE OM** (PANAGOLA) | 3.00 | 2.67 | 3.00 | 2.75 | 2.14 | 2.33 | 2.33 | 2.00 | 1.00 | 2.00 |
| Mobile | Mental Health | **Natural Stress Relief Hypnosis** (Mastermind App) [a] | 3.67 | 2.33 | 2.25 | 1.75 | 1.71 | 2.33 | 1.00 | 1.00 | 1.00 | 4.00 |
| Mobile | Mental Health | **Omvana** (Mindvalley LLC) [b] | 3.67 | 4.33 | 3.00 | 3.25 | 2.00 | 3.33 | 3.33 | 3.00 | 1.00 | 4.00 |
| Mobile | Mental Health | **PPD Gone** (EasyProApps.com - blue logo) | 2.33 | 2.33 | 1.25 | 2.00 | 1.00 | 1.33 | 1.00 | 2.00 | 1.00 | 4.00 |
| Mobile | Mental Health | **Qi Gong Meditation Relaxation** (Excel At Life) | 3.33 | 2.33 | 2.40 | 3.25 | 1.57 | 2.67 | 3.00 | 4.00 | 1.00 | 3.00 |
| Mobile | Mental Health | **Sleep Genius With Alarm** (Sleep Genius) [b] | 2.67 | 3.67 | 2.40 | 2.75 | 2.29 | 1.67 | 2.33 | 4.00 | 1.00 | 4.00 |
| Mobile | Mental Health | **SuperBetter** (SuperBetter, LLC) | 2.33 | 3.67 | 3.00 | 3.00 | 3.29 | 3.00 | 2.33 | 3.00 | 3.00 | 4.00 |
| Mobile | Mental Health | **Toxic thinking** (boultons MultiMedia) | 3.00 | 1.67 | 1.00 | 1.25 | 1.00 | 1.00 | 1.00 | 3.00 | 1.00 | 4.00 |
| Mobile | Mental Health | **WellMind** (Blue Step Solutions) | 4.00 | 4.33 | 3.00 | 3.00 | 2.29 | 2.67 | 2.33 | 2.00 | 1.00 | 4.00 |
| Mobile | Mental Health | **Wildflowers Mindfulness** (Mobio Interactive Inc.) [b] | 4.00 | 4.33 | 3.60 | 4.00 | 3.14 | 3.00 | 2.00 | 2.00 | 1.00 | 2.00 |
| Mobile | Mental Health | **Worry Box** (Excel At Life) | 2.33 | 2.33 | 2.20 | 1.50 | 1.43 | 1.67 | 1.33 | 4.00 | 1.00 | 2.00 |
| Mobile | Mental Health | **Yoga Helps Relieve Depression** (Apps Viva) [a] | 4.00 | 1.67 | 1.00 | 1.00 | 1.14 | 1.00 | 1.00 | 1.00 | 1.00 | 4.00 |
| Website | Health Related Behaviors | **14 Weeks to a Healthier You** (http://www.nchpad.org/14weeks/index.php) | 3.33 | 3.00 | 3.60 | 4.25 | 3.29 | 3.67 | 3.67 | 4.00 | 2.00 | 2.00 |

| Delivery | Clinical Aim | Name | Usability | Visual Design | UE[c] | Content | TP[c] | TA[c] | General Subjective Evaluation | Credibility | Evidence Based Program | Privacy Checklist |
|---|---|---|---|---|---|---|---|---|---|---|---|---|
| Website | Health Related Behaviors | **American Council for Exercise** (https://www.acefitness.org/acefit/fitness_programs_article.aspx?ID=3159) [d] | 3.33 | 2.33 | 1.75 | 3.25 | 1.57 | 1.33 | 1.67 | 6.00 | 1.00 | 3.00 |
| Website | Health Related Behaviors | **Become an Ex** (http://www.becomeanex.org/login.php) | 2.67 | 2.00 | 3.60 | 4.25 | 3.29 | 3.00 | 3.67 | 4.00 | 2.00 | 3.00 |
| Website | Health Related Behaviors | **Breath Better** (https://www.breathebetter.me/BreatheBetterLogin.aspx) | 3.67 | 2.67 | 2.80 | 4.00 | 2.43 | 2.33 | 2.67 | 3.00 | 1.00 | 2.00 |
| Website | Health Related Behaviors | **Control Your Drinking Online** (http://www.acar.net.au/control_your_drinking_online.html) | 2.67 | 1.33 | 1.80 | 4.50 | 2.00 | 2.00 | 2.00 | 5.00 | 1.00 | 3.00 |
| Website | Health Related Behaviors | **Darebee** (http://darebee.com/programs/foundation-light-program.html?showall=&start=1) [d] | 1.33 | 1.67 | 1.80 | 2.75 | 1.57 | 1.33 | 1.67 | 3.00 | 1.00 | 4.00 |
| Website | Health Related Behaviors | **Do Yoga With Me** (https://www.doyogawithme.com/yoga_programs) [d] | 3.33 | 4.00 | 3.40 | 4.25 | 2.43 | 3.33 | 3.67 | 4.00 | 1.00 | 5.00 |
| Website | Health Related Behaviors | **Down Your Drink** (https://www.downyourdrink.org.uk/session/new) | 2.33 | 2.67 | 3.00 | 4.25 | 2.00 | 2.00 | 1.67 | 5.00 | 2.00 | 3.00 |
| Website | Health Related Behaviors | **Eat This Much** (www.eatthismuch.com) | 3.33 | 4.00 | 3.60 | 3.25 | 2.29 | 1.67 | 1.67 | 3.00 | 1.00 | 2.00 |
| Website | Health Related Behaviors | **Everyday Health** (https://www.everydayhealth.com/login/) | 2.00 | 2.33 | 1.80 | 2.00 | 2.29 | 1.33 | 1.33 | 5.00 | 1.00 | 1.00 |

| Delivery | Clinical Aim | Name | Usability | Visual Design | UE[C] | Content | TP[C] | TA[C] | General Subjective Evaluation | Credibility | Evidence Based Program | Privacy Checklist |
|---|---|---|---|---|---|---|---|---|---|---|---|---|
| Website | Health Related Behaviors | **Fitness.com** (http://www.fitness.com/login.php?ref=%2F) [d] | 2.67 | 2.00 | 2.20 | 2.25 | 1.43 | 2.00 | 1.33 | 4.00 | 1.00 | 5.00 |
| Website | Health Related Behaviors | **Free Trainers** (https://www.freetrainers.com/account/login/) [d] | 2.33 | 2.33 | 2.00 | 2.75 | 1.57 | 2.00 | 1.33 | 3.00 | 1.00 | 3.00 |
| Website | Health Related Behaviors | **Healthy Life Project** (http://www.healthylifeproject.com.au/program/web/dashboard) | 3.00 | 3.67 | 2.80 | 3.25 | 2.71 | 2.00 | 2.00 | 2.00 | 1.00 | 4.00 |
| Website | Health Related Behaviors | **My Fitness Pal** (https://www.myfitnesspal.com/) | 4.00 | 2.67 | 3.40 | 3.50 | 2.71 | 2.67 | 2.00 | 5.00 | 2.00 | 3.00 |
| Website | Health Related Behaviors | **My Healthy Balance** (https://myhealthybalance.com.au/Account/Login) [d] | 3.33 | 4.00 | 3.80 | 4.00 | 3.43 | 2.67 | 3.33 | 2.00 | 1.00 | 1.00 |
| Website | Health Related Behaviors | **My Personal Home Trainer** (http://www.myhomepersonaltrainer.com/home/member-login.asp) [d] | 2.33 | 2.00 | 2.00 | 2.25 | 1.71 | 1.33 | 1.33 | 2.00 | 1.00 | 6.00 |
| Website | Health Related Behaviors | **Quit Coach** (http://www.quitcoach.org.au/) | 2.67 | 2.00 | 2.00 | 3.25 | 2.00 | 2.00 | 1.67 | 4.00 | 3.00 | 1.00 |
| Website | Health Related Behaviors | **SmokeFree** (https://smokefree.gov/build-your-quit-plan) [d] | 4.33 | 3.00 | 2.50 | 2.50 | 1.86 | 1.33 | 2.33 | 4.00 | 2.00 | 1.00 |
| Website | Health Related Behaviors | **Start Your Diet** (http://www.startyourdiet.com/login.php) | 2.33 | 2.00 | 2.80 | 2.75 | 2.57 | 1.67 | 1.67 | 2.00 | 1.00 | 5.00 |
| Website | Health Related Behaviors | **Vet Change** (https://vetchange.org/account/login) | 3.67 | 3.67 | 3.60 | 4.50 | 3.14 | 3.00 | 3.33 | 5.00 | 3.00 | 1.00 |

| Delivery | Clinical Aim | Name | Usability | Visual Design | UE[c] | Content | TP[c] | TA[c] | General Subjective Evaluation | Credibility | Evidence Based Program | Privacy Checklist |
|---|---|---|---|---|---|---|---|---|---|---|---|---|
| Website | Health Related Behaviors | **Your First Step To** Change(http://s96539219.onlinehome.us/toolkits/alcohol/main.htm) | 3.33 | 2.00 | 2.00 | 3.25 | 1.57 | 1.67 | 1.33 | 3.00 | 1.00 | 1.00 |
| Website | Mental Health | **Calm** (https://www.calm.com/login?source=homepage) | 4.00 | 4.67 | 3.40 | 4.00 | 3.29 | 3.67 | 4.00 | 3.00 | 1.00 | 1.00 |
| Website | Mental Health | **Daily Challenge** (https://account.meyouhealth.com/dailychallenge/session/new) | 4.00 | 2.67 | 3.60 | 4.00 | 3.43 | 3.00 | 4.00 | 6.00 | 3.00 | 2.00 |
| Website | Mental Health | **Depression Center** (http://www.depressioncenter.net/) | 3.33 | 2.33 | 2.50 | 4.25 | 2.71 | 2.33 | 2.00 | 3.00 | 1.00 | 4.00 |
| Website | Mental Health | **Depression ToolKit** (http://www.depressiontoolkit.org/stressgym/) [d] | 3.33 | 2.00 | 1.25 | 3.50 | 1.57 | 1.67 | 1.00 | 6.00 | 1.00 | 0.00 |
| Website | Mental Health | **E-Couch** (https://ecouch.anu.edu.au/ecouch/dep_launch) | 3.33 | 2.67 | 3.50 | 4.50 | 2.86 | 3.33 | 3.33 | 5.00 | 3.00 | 0.00 |
| Website | Mental Health | **Finding Optimism** (https://www.optimismonline.com/login.php) | 2.67 | 2.00 | 3.00 | 3.25 | 2.71 | 1.33 | 1.33 | 3.00 | 1.00 | 1.00 |
| Website | Mental Health | **Living Life to The Fullest** (https://www.llttf.com/index.php?section=page&page_seq=12) [d] | 2.67 | 2.33 | 2.00 | 3.50 | 2.00 | 2.00 | 1.67 | 5.00 | 3.00 | 2.00 |
| Website | Mental Health | **Mental Health Online** (https://www.mentalhealthonline.org.au/pages/login) | 3.00 | 2.33 | 2.40 | 4.25 | 2.14 | 2.67 | 1.67 | 5.00 | 2.00 | 1.00 |
| Website | Mental Health | **MoodGYM** (https://moodgym.anu.edu.au/welcome) | 3.67 | 2.00 | 2.40 | 3.75 | 2.14 | 2.67 | 2.00 | 6.00 | 2.00 | 0.00 |

| Delivery | Clinical Aim | Name | Usability | Visual Design | UE[c] | Content | TP[c] | TA[c] | General Subjective Evaluation | Credibility | Evidence Based Program | Privacy Checklist |
|---|---|---|---|---|---|---|---|---|---|---|---|---|
| Website | Mental Health | **My Compass** (https://www.mycompass.org.au/) | 4.00 | 2.67 | 3.20 | 4.75 | 2.71 | 3.00 | 2.33 | 5.00 | 4.00 | 1.00 |
| Website | Mental Health | **OnTrack** (https://www.ontrack.org.au/site/login) | 4.00 | 2.67 | 3.40 | 3.75 | 2.86 | 3.33 | 2.33 | 5.00 | 2.00 | 1.00 |
| Website | Mental Health | **Palouse Mindfulness** (http://palousemindfulness.com/selfguidedMBSR_week1.html)[d] | 2.67 | 2.00 | 3.20 | 3.50 | 2.14 | 3.00 | 2.67 | 4.00 | 1.00 | 1.00 |
| Website | Mental Health | **Panic Center** (http://www.paniccenter.net/Program/Toolbox.aspx) | 3.00 | 2.33 | 2.50 | 3.75 | 2.29 | 2.67 | 2.00 | 3.00 | 2.00 | 3.00 |
| Website | Mental Health | **Peer Leadership Center** (http://www.peerleadershipcenter.org/assnfe/CourseOnline.asp?MODE=VIEW&encEvaId=&crsId=4&modId=15&fromSum=1) [d] | 3.33 | 3.00 | 2.20 | 3.50 | 1.86 | 2.67 | 1.67 | 5.00 | 1.00 | 4.00 |
| Website | Mental Health | **Serene** (http://serene.me.uk/kiosk-0/anxiety_menu.php) [d] | 3.67 | 2.33 | 2.00 | 3.00 | 1.43 | 1.67 | 1.00 | 3.00 | 2.00 | 0.00 |
| Website | Mental Health | **Smiling Mind** (//app.smilingmind.com.au/programs/7/) | 3.00 | 3.00 | 3.40 | 4.00 | 2.71 | 3.00 | 3.00 | 5.00 | 1.00 | 1.00 |
| Website | Mental Health | **Start 2** (http://www.start2.co.uk/)[d] | 2.67 | 2.33 | 2.80 | 3.50 | 2.29 | 2.67 | 2.67 | 3.00 | 1.00 | 3.00 |
| Website | Mental Health | **Stop Breathe Think** (https://app.stopbreathethink.org/) | 4.00 | 4.67 | 3.80 | 4.00 | 3.14 | 3.33 | 3.67 | 3.00 | 1.00 | 1.00 |
| Website | Mental Health | **The Desk** (https://www.thedesk.org.au/login?login) | 4.00 | 3.67 | 4.00 | 3.75 | 2.14 | 3.00 | 4.00 | 5.00 | 2.00 | 6.00 |

| Delivery | Clinical Aim | Name | Usability | Visual Design | UE[C] | Content | TP[C] | TA[C] | General Subjective Evaluation | Credibility | Evidence Based Program | Privacy Checklist |
|---|---|---|---|---|---|---|---|---|---|---|---|---|
| Website | Mental Health | **The Journal** (https://depression.org.nz/is-it-depression-anxiety/self-test/depression-test/) | 3.33 | 3.33 | 3.60 | 4.25 | 3.29 | 3.67 | 3.33 | 5.00 | 1.00 | 2.00 |
| Website | Mental Health | **Youth Mental Health** (http://ymhonline.com/loginpage.asp) | 3.33 | 2.33 | 2.20 | 3.00 | 1.29 | 2.00 | 1.33 | 5.00 | 1.00 | 0.00 |

Notes: [a] These mobile applications were identified by the raters to receive high Usability scores only since they were very lean and therefore easy to learn and use regardless of design aspects that enhance usability. [b] This free to use intervention program also includes the ability to access a paid version that was not examined. [C] UE: User Engagement; TP: Therapeutic Persuasiveness; TA: Therapeutic Alliance. [d] This web-based program was identified to “nest” within a larger website, in which, high percentage of users were expected to access the website for different reasons than the intervention program itself.
