# Supplementary material for: Enlight: A Comprehensive Quality and Therapeutic Potential Evaluation Tool for Mobile and Web-Based eHealth Interventions
Source: J Med Internet Res. 2017 Mar 21;19(3):e82. doi: 10.2196/jmir.7270 (PMC5380814; doi:10.2196/jmir.7270)
Supplement: Multimedia Appendix 8 [file jmir_v19i3e82_app8.pdf]

Multimedia Appendix 8 - Descriptive Statistics of Quality Section Items

Means and Standard Deviations of Scale items and Constructs by Program's Delivery Mean and Clinical Aim.

Items and Constructs	Delivery Mean				Clinical Aim					
	Mobile app (n=42)		Web-based (n=42)		Healthy Behaviors (n=42)		Mental Health (n=42)		Total (N=84)	
	M	SD	M	SD	M	SD	M	SD	M	SD
**Usability**										
Navigation	3.21	0.84	3.02	0.81	3.14	0.90	3.10	0.76	3.12	0.83
Learnability	3.79	0.75	3.43	0.77	3.52	0.89	3.69	0.64	3.61	0.78
Ease of Use	3.38	0.83	3.05	0.73	3.19	0.89	3.24	0.69	3.21	0.79
**Visual Design**										
Aesthetic Design	2.69	0.95	2.86	0.98	2.79	0.87	2.76	1.05	2.77	0.96
Layout	2.74	0.91	2.62	0.76	2.67	0.82	2.69	0.87	2.68	0.84
Size	3.36	0.93	2.57	0.94	2.93	0.97	3.00	1.06	2.96	1.01
**User Engagement**										
Content Presentation	2.43	0.94	2.71	0.97	2.60	1.04	2.55	0.89	2.57	0.96
Interactive	2.59	1.21	2.67	0.85	2.62	1.08	2.65	0.98	2.63	1.03
Not Irritating	2.55	0.89	2.94	1.03	2.78	0.98	2.72	0.99	2.75	0.98
TTP	2.52	0.92	2.98	0.92	2.81	0.92	2.69	0.98	2.75	0.94
Captivating	2.38	0.91	2.69	0.87	2.52	0.83	2.55	0.97	2.54	0.90
**Content**										
Evidence Based Content	2.48	0.99	4.26	0.91	3.14	1.24	3.60	1.34	3.37	1.31
Information Provision Quality	2.52	1.15	3.55	0.92	3.00	1.08	3.07	1.24	3.04	1.16
Complete and Concise	2.12	0.97	3.14	0.87	2.62	0.96	2.64	1.14	2.63	1.05
Clarity about the Program	2.50	0.77	3.41	0.80	2.86	0.90	3.05	0.91	2.95	0.90

	Delivery Mean				Clinical Aim					
	**Mobile app (n=42)**		**Web-based (n=42)**		**Healthy Behaviors (n=42)**		**Mental Health (n=42)**		**Total (N=84)**	
	M	SD	M	SD	M	SD	M	SD	M	SD
**Therapeutic Persuasiveness**										
Call for Action	2.21	0.81	2.57	0.74	2.55	0.74	2.24	0.82	2.39	0.79
Load Reduction of Therapeutic Goals	2.14	1.07	2.48	0.80	2.14	0.95	2.48	0.94	2.31	0.96
Therapeutic Rational/Pathway	2.29	0.92	2.76	0.82	2.55	0.89	2.50	0.92	2.52	0.90
Rewards for Meeting Goals	1.79	0.98	1.86	0.90	1.95	0.96	1.69	0.90	1.82	0.93
Data Driven/Adaptive	1.81	0.92	1.88	0.74	1.93	0.84	1.76	0.82	1.85	0.83
Ongoing Feedback	2.02	1.05	2.05	0.88	2.17	0.99	1.91	0.93	2.04	0.96
Expectations and Relevance	2.48	0.80	2.88	0.89	2.64	0.93	2.71	0.81	2.68	0.87
**Therapeutic Alliance**										
Basic Acceptance and Support	2.24	0.76	2.60	0.80	2.31	0.75	2.52	0.83	2.42	.79
Confidence, Positive Therapeutic Expectations	1.98	0.90	2.64	0.91	2.19	0.94	2.43	0.97	2.31	0.96
Relatedness	1.76	0.85	1.98	0.87	1.60	0.83	2.14	0.81	1.87	0.86
**General Subjective Evaluation**										
Appropriate Features to Meet the Clinical Aim	2.00	0.94	2.55	0.97	2.21	0.95	2.33	1.03	2.27	0.99
Right Mix of Ability Vs. Motivation	1.74	0.83	1.93	0.75	1.79	0.75	1.88	0.83	1.83	0.79
I like the program.	1.93	1.05	2.41	1.29	2.10	1.14	2.24	1.25	2.17	1.19

notes: M = Mean; SD = Standard Deviation.
