# Supplementary material for: Enlight: A Comprehensive Quality and Therapeutic Potential Evaluation Tool for Mobile and Web-Based eHealth Interventions
Source: J Med Internet Res. 2017 Mar 21;19(3):e82. doi: 10.2196/jmir.7270 (PMC5380814; doi:10.2196/jmir.7270)
Supplement: Multimedia Appendix 9 [file jmir_v19i3e82_app9.pdf]

Multimedia Appendix 9 – Kappa Reliability Scores of Credibility Checklist and Privacy Explanation Checklist Items

Inter-raters Agreement (Kappa) on Categorical Items that Assemble the Credibility Checklist

	Owner's Credibility	Maintenance	Strong Advisory Support	Evidence for Successful Implementation
**Total (n=84)**				
Kappa	.90	- [a]	.82	1.00
(95% CI)	(.81-1.00)	-	(.70-.94)	(1.00-1.00)
**Mobile (n=42)**				
Kappa	.87	.95	.79	1.00
(95% CI)	(.69-1.05)	(.85-1.00)	(.58-1.00)	(1.00-1.00)
**Website (n=42)**				
Kappa	.90	- [a]	.81	1.00
(95% CI)	(.76-1.03)	-	(.65-.96)	(1.00-1.00)
**Mental Health (n=42)**				
Kappa	.81	1.00 [b]	.82	1.00
(95% CI)	(.63-.99)	(1.00-1.00)	(.67-.97)	(1.00-1.00)
**Healthy Behaviors (n=42)**				
Kappa	1.00	.90 [b]	.76	1.00
(95% CI)	(1.00-1.00)	(.71-1.00)	(.51-1.00)	(1.00-1.00)

notes: Third Party Endorsement was not calculated since none of the programs was endorsed - reliability calculations are not applicable on constant measures.

[a] Maintenance was not evaluated for web-based programs and therefore reliability scores appear for mobile applications and separately by clinical aim. [b] Based on n=21 examined mobile applications.

Inter-raters Agreement (Kappa) on Categorical Items that Assemble the Privacy Explanation Checklist

	The system informs users of the data journey in detail to understand all sources of data exposure.	The system explicitly notifies how personal health information and/or personal identifiers will **be kept confidential** unless clear permission was given.	The system explicitly notifies how personal information and/or personal identifiers **may be used** before data is collected.	The system tunnels users through the terms of use explicitly.	The system allows users to keep identifiers private.	When not apparent the system lets users know when they go public.	The system warns the users from providing private information and asks permission to provide such information when applicable.
**Total (n=84)**							
Kappa	.95	.98	.95	.70	1.00	1.00	.97
(95% CI)	(.88-1.00)	(.93-1.00)	(.89-1.00)	(.46-.95)	(1.00-1.00)	(1.00-1.00)	(.90-1.00)
**Mobile (n=42)**							
Kappa	.91	1.00	.85	.64	1.00	1.00	1.00
(95% CI)	(.73-1.00)	(1.00-1.00)	(.64-1.00)	(.19-1.00)	(1.00-1.00)	(1.00-1.00)	(1.00-1.00)
**Website (n=42)**							
Kappa	.95	.94	1.00	.73	1.00	- [a]	.95
(95% CI)	(.84-1.00)	(.82-1.00)	(1.00-1.00)	(.44-1.00)	(1.00-1.00)	-	(0.85-1.00)
**Mental Health (n=42)**							
Kappa	.95	1.00	.90	.69	1.00	1.00	1.00
(95% CI)	(.84-1.00)	(1.00-1.00)	(.77-1.00)	(.41-.97)	(1.00-1.00)	(1.00-1.00)	(1.00-1.00)
**Healthy Behaviors (n=42)**							
Kappa	.95	.95	1.00	.66	1.00	- [a]	.94
(95% CI)	(.85-1.00)	(.86-1.00)	(1.00-1.00)	(.03-1.00)	(1.00-1.00)	-	(.83-1.00)

notes: [a] In these cases 100% agreement was noted, but Kappa was not calculated since all of these programs met criteria (constant result).

Interpretation

Based on criteria established by Landis and Koch (1977) the strength of agreement between raters was mostly at the outstanding agreement range (Kappa >.80; 44/51 ratings, 86.3%) with the minority of scores being at the substantial agreement range (.60 < Kappa < .80; 7/51 ratings, 13.7%).

Reference

Landis, J. R., & Koch, G. G. (1977). The measurement of observer agreement for categorical data. *biometrics*, 159-174.
